# PepT1-targeted nanodrug based on co-assembly of anti-inflammatory peptide and immunosuppressant for combined treatment of acute and chronic DSS-induced ColitiS

**DOI:** 10.3389/fphar.2024.1442876

**Published:** 2024-08-15

**Authors:** Daifang Zhang, Longqi Jiang, Fengxu Yu, Pijun Yan, Yong Liu, Ya Wu, Xi Yang

**Affiliations:** ^1^ Department of Cardiovascular Surgery, The Affiliated Hospital of Southwest Medical University, Luzhou, China; ^2^ Metabolic Vascular Disease Key Laboratory of Sichuan Province, The Affiliated Hospital of Southwest Medical University, Luzhou, China; ^3^ Department of Vascular Surgery, The Affiliated Hospital of Southwest Medical University, Luzhou, China; ^4^ Department of Endocrinology and Metabolism, The Affiliated Hospital of Southwest Medical University, Luzhou, Sichuan, China; ^5^ Key Laboratory of Medical Electrophysiology, Ministry of Education & Medical Electrophysiological Key Laboratory of Sichuan Province (Collaborative Innovation Center for Prevention of Cardiovascular Diseases), Institute of Cardiovascular Research, Southwest Medical University, Luzhou, China

**Keywords:** colitis, PepT1-targeting, combined treatment, KPV, FK506

## Abstract

**Introduction:**

Inflammatory bowel disease (IBD), as a chronic and recurrent inflammatory bowel diseases with limited therapeutic outcomes, is characterized by immune disorders and intestinal barrier dysfunction. Currently, the most medications used to cure IBD in clinic just temporarily induce and maintain remission with poor response rates and limited outcomes. Therefore, it is urgently necessary to develop an appropriate therapeutic candidate with preferable efficacy and less adverse reaction for curing IBD.

**Methods:**

Five groups of mice were utilized: control that received saline, DSS group (mice received 2.5% DSS or 4% DSS), KPV group (mice received KPV), FK506 group (mice received FK506) and NPs groups (mice received NPs). The effect of NP on the inflammatory factors of macrophage was evaluated using CCK-8, quantitative polymerase chain reaction (PCR), Elisa and Western blot (WB). Immunofluorescent staining revealed the targeting relationship between NP and Petp-1. Immunohistochemistry staining showed the effect of NP on tight junction proteins. Moreover, in vivo animal experiments confirmed that NPs reduced inflammatory levels in IBD.

**Results and Discussion:**

After administering with NPs, mice with DSS-induced acute or chronic colitis exhibited significant improvement in body weight, colon length, and disease activity index, decreased the level of the factors associated with oxidative stress (MPO, NO and ROS) and the inflammatory cytokines (TNF-α, IL-1β and IL-6), which implied that NPs could ameliorate murine colitis effectively. Furthermore, treating by NPs revealed a notable reduction of the expressions of CD68 and CD3, restoring the expression levels of tight junction proteins (Claudin-5, Occludin-1, and ZO-1) were significantly restored, surpassing those observed in the KPV and FK506 groups. which indicated that NPs can reduce inflammation and enhance epithelial barrier integrity by decreasing the infiltration of macrophages and T-lymphocytes. Collectively, those results demonstrated the effectively therapeutic outcome after using NPs in both acute and chronic colitis, suggesting that the newly co-assembled of NPs can be as a potential therapeutic candidate for colitis.

## 1 Introduction

Inflammatory bowel disease (IBD) is an idiopathic, chronic and recurrent inflammation disorder of the gastrointestinal tract, which not only affects patients’ quality of life, but also imposes a significant burden on public healthcare (Colombel et al., 2004; Lamb et al., 2019). As a global disease, IBD is prevalent in North America and Europe, particularly in developed and urbanized areas, and the incidence of IBD in developing countries has risen dramatically in the past decade, such that it is predicted there will be more than 1.5 million IBD patients in China by 2025 (Xavier and Podolsky, 2007; Chang, 2020). Currently, the most widely used medications in clinic to manage IBD contain anti-inflammatory drugs (aminosalicylates and corticosteroids), immunosuppressants, and antibiotics (Kaplan, 2015; Malik, 2015). However, these agents just temporarily induce and maintain remission with poor response rates and limited outcomes (Grossberg et al., 2022). Additionally, they also bring undesirable side events, such as infections, infusion-related reactions, and possibly cancer risk (Peyrin-Biroulet and Lemann, 2011). Therefore, it is urgently necessary to develop an appropriate therapeutic candidate with preferable efficacy and less adverse reaction for clinical transformation (Lamb et al., 2022).

Colitis, as a chronic and recurrent inflammatory bowel diseases with limited therapeutic outcomes, is characterized by immune disorders and intestinal barrier dysfunction. Currently, a wide variety of colon-targeted delivery systems have been developed to treat IBD to overcome the above challenges, while these systems often require carrier materials which cause low loading efficiency of drugs, potential long-term toxicities and complex preparation processes, which restrict their clinical translation (dos Santos et al., 2021; Cui et al., 2021; Kotla et al., 2019). Fortunately, carrier-free nanodrug as a newly- promising platform has been widely utilized. However, there still remain some issues that needed to pay more attention for designing the ideal carrier-free nanodrugs, including how to select appropriate drug self-assembly and how to increase the concentration of the nanodrug at the lesion site, etc.

Tacrolimus (FK506) is an immunosuppressive macrolide isolated, which could treat IBD though effectively suppressing the effect of T-lymphocytes and inhibiting IL-2 expression (Ogata et al., 2006; Regmi et al., 2019). However, previously reported patients with IBD treated with oral FK506 may suffer from renal function damage and infectious complications, including bacterial pneumonia, meningitis, and CMV infection (Thin et al., 2013). Thus, it is necessary to deliver FK506 to colitis for balancing the effect and toxicity of FK506, which requires that the co-assembled drugs possess the ability to target colitis. Studies have revealed that colonic epithelial cells and macrophages overexpress PepT1 in colitis (Charrier et al., 2006; Ayyadurai et al., 2013). KPV is a tripeptide (Lys-Pro-Val) which possesses anti-inflammatory properties, displaying a high affinity with PepT1, meanwhile, study has been shown that its anti-inflammatory properties contribute significantly towards the restoration of intestinal barrier dysfunction (Merlin et al., 2001; Ayyadurai et al., 2013). Considering the characteristics of immune disorders and intestinal barrier dysfunction in colitis, combined with our previous study (Wu et al., 2019a; Wang et al., 2023), we hypothesized that the nanoparticles based on the co-assembly of an anti-inflammatory peptide (KPV) and an immunosuppressant (FK506) can better treat IBD.

**SCHEME 1:**
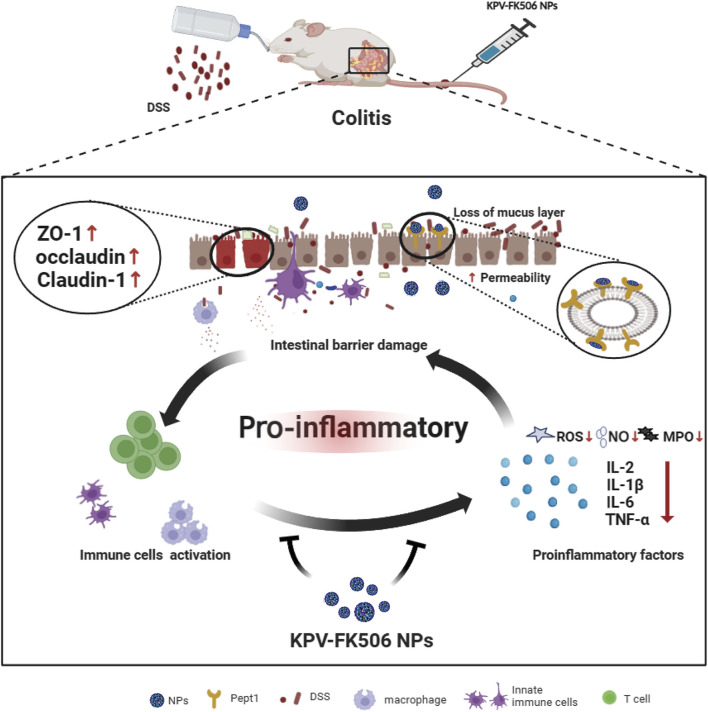
Illustration of NPs on colitis.

Herein, this study aimed to develop the PepT1-medied NPs targeting nanodrug for relieving colitis. As shown in Scheme 1, we interestingly observed that the NPs accumulated in the diseased region by recognizing the abnormally expressed PepT1 in colitis after administration. On the one hand, NPs would alleviate the inflammatory response by reducing the expression of intestinal inflammatory factors, NO, ROS, and MPO. On the other hand, NPs would enhance epithelial barrier integrity to relieve colitis by increasing intestinal Mucin-2, ZO-1, and Claudin5 expression. Our study confirmed that the newly co-assembled of NPs could be a therapeutic target for the treatment of colonic inflammation.

## 2 Materials and methods

### 2.1 Materials

Tacrolimus (FK506) was purchased from MCE (NJ, United States). Lysine-proline-valine (KPV, ≥99%, MW = 342.40 Da) was obtained from GL Bio-Chem Co., Ltd. (Shanghai, China). 1,2-Distearoyl-sn-glycero-3-phosphoethanolamine-N [methoxy (polyethylene glycol)-2000] (DSPE-PEG2000) was from Advanced Vehicle Technology Co., Ltd. (Shanghai, China). Lipopolysaccharides (LPS, ≥98%), Fetal bovine serum (FBS) and RPMI Medium 1640 were provided by Sigma-Aldrich (MO, United States). Dextran sulfate sodium (DSS, MW = 36 kDa–50 kDa) was supplied by MP Biomedical (CA, United States). RNA extraction reagent was obtained from Toyobo (Osaka, Japan). RNA reverse transcription kit was purchased from Vazyme (Nanjing, China). ELISA kits (TNF-α, IL-1β, IL-6, IL-2, IFN-γ, MPO, NO, ROS, AST, ALT, BUN, CRE) were supplied by Nanjing Jiancheng Biotechnology Co., Ltd. (Nanjing, China). Rabbit IL-1β antibody and rabbit IL-2 antibody were purchased from CST (Boston, United States). Rabbit β-actin antibody, PepT1, CD68, CD3, Occludin-1, claudin-5, ZO-1 were obtained from Affinity Biosciences (Cincinnati, United States). HRP-labeled Goat Anti-Rabbit IgG (H + L) was purchased from EpiZyme Bio-pharmaceutical Technology Co., Ltd. (Shanghai, China).

### 2.2 Cells and animals

RAW264.7, NCM460 and Caco-2 cell lines were supplied by American Type Culture Collection (ATCC, Shanghai, China). Male C57BL/6 mice (8 weeks, 22 ± 2 g) were obtained from Beijing Huafukang Biotechnology (Beijing, China), housed in a room under constant temperature (20°C ± 2°C) and relative humidity (55–70%) with a 12-h light/dark cycle. The mice were randomly divided into several groups with 1-week acclimatization before the experiment. Animal experiments were performed following the Institutional Animal Care and Use Committee (IACUC) guidelines and were approved by the Animal Ethics Committee of Southwest Medical University.

### 2.3 Preparation of the NPs

The NPs were prepared using a dialysis method. Briefly, 3 mg of KPV was dissolved in 3 mL of double distilled water, and the solution was adjusted to a pH of approximately 9.0 using 0.4 M NaOH. Next, 400 μL of FK506 solution (2 mg/mL) and 0.12 mg of DSPE-PEG2000 dissolved in DMSO were added dropwise to the KPV solution. The mixture was then transferred to a dialysis bag and the water was changed every hour. After 24 h of dialysis, samples were collected by centrifugation at 15,000 rpm and washed three times with double distilled water. The samples were stored in the dark at 4°C for future use.

### 2.4 Characterization of the NPs

The average hydrodynamic diameters and zeta potential were measured by ZetaPlus Zeta Potential Analyzer (Brookhaven Instrument Corporation). The NPs were evenly dispersed in distilled water for detection at 658 nm of laser excitation and 90° of dynamic light scattering angle. The morphology of NPs were analyzed by transmission electron microscopy (Thermo, MA, United States). Diluted NPs suspension was deposited onto copper grids for a period of time until air-dried before analysis. The FK506 content was determined by HPLC using an Agilent 1260 instrument (Agilent, Palo Alto, United States) at 210 nm on a Diamonsil C18 (5 μm, 250 × 4.6 mm) with a mobile phase consisting of acetonitrile and 0.1% phosphoric acid (80:20) at a flow rate of 1.0 mL/min. The hemolysis test was conducted in accordance with prior reports for the hemocompatibility assay. To prepare erythrocytes for suspension in normal physiological saline, they were collected from healthy C57BL/6 mice and washed three times with normal saline. Then, a centrifuge tube was filled with erythrocyte suspension and nanoparticles solution. As controls, we used PBS as the negative one and FK506 as the positive one. After being incubated at 37°C for 1 h, the samples were spun at 5,000 rpm for 10 min. Then, 100 uL of the supernatant from each tube was carefully collected and added to each well of 96-well plates in sextuplicate. The percentage of red blood cell hemolysis was determined by measuring the absorbance of each well at 540 nm using a microplate reader. The formula for this percentage is (percentage of lysis % = (OD_test_ − OD_negative_)/OD _positive_ × 100) (n = 6) (Shi et al., 2015).

### 2.5 Cell viability assay

The cytotoxicity of the NPs to macrophages was measured by a standard CCK8 assay. RAW264.7 cells were cultured with different concentrations of KPV, FK506, and NPs for 24 h, followed by CCK8 procedures. The absorbance at 450 nm was recorded by a microreader (FLUOstar Omega, BMG LABTECH, Offenburg, GER). The levels of cytokines (TNF-α, IL-1β, IL-6, IL-2) were measured using an ELISA kit (Jiancheng, Nanjing, China) following the manufacturer’s protocol.

### 2.6 Cytokine analysis by qPCR

Total RNA was extracted from colonic tissues using TRIzol reagent (Invitrogen, Carlsbad, CA, United States) according to the manufacturer’s instructions. The yield and quality of RNA were verified with a NanoDrop 2000 (Thermo Scientific, Waltham, MA, United States). cDNA was synthesized from RNA using ReverTra Ace^®^ qPCR RT Master Mix (Toyobo, Osaka, Japan). mRNA expression was quantified by quantitative real-time reverse-transcription polymerase chain reaction using ChamQ Universal SYBR qPCR Master Mix. Results were analyzed using the 2^−ΔΔCT^ method. The primer sequences of the rats were designed as follows:

TNF-α forward, 5′-ATG​TCT​CAG​CCT​CTT​CTC​ATT​C-3′
TNF-α reverse, 5′-GCT​TGT​CAC​TCG​AAT​TTT​GAG​A-3′;
IL-1β forward, 5′-CTG​CAG​CTG​GAG​AGT​GTG​GA-3′,
IL-1β reverse, 5′-TGC​TCT​GCT​TGT​GAG​GTG​CT-3′;
IL-2 forward, 5′-AGC​TCG​CAT​CCT​GTG​TCA​CAT​TG-3′,
IL-2 reverse, 5′-CTG​CTG​TGC​TTC​CGC​TGT​AGA​G-3′;
IL-6 forward, 5′-CTC​CCA​ACA​GAC​CTG​TCT​ATA​C-3′,
IL-6 reverse, 5′-CCA​TTG​CAC​AAC​TCT​TTT​CTC​A-3′;
IFN-γ forward, 5′-CTG​GAG​GAA​CTG​GCA​AAA​GGA​TGG-3′,
IFN-γ reverse, 5′-GAC​GCT​TAT​GTT​GTT​GCT​GAT​GGC-3′.

### 2.7 Western blot assay

Total protein was extracted from fresh colon tissues and quantified by the BCA Protein Assay Kit. Next, the extracted proteins were boiled in a loading buffer, separated by 12.5% SDS-PAGE, and transferred onto polyvinylidene fluoride membranes (PVDF, 0.22 μm, Millipore, Germany). After blocking with 5% BSA solution for 1 h, the PVDF membranes were incubated with primary antibodies overnight at 4°C, followed by incubation with secondary antibodies for 1.5 h at room temperature. The blots were then visualized using the Omni-ECL™Femto Light Chemiluminescence Kit (EpiZyme, China). Densitometry quantifications were performed using the software VisionWorksLS (Chemstudio-815).

### 2.8 DSS-induced colitis in mice

The colitis model was established according to a previous method with slight modifications (Wirtz et al., 2017; Xue et al., 2018). Acute colitis was induced in male C57BL/6 mice by free drinking water with 4% DSS for 7 days, while the mice received drug therapy every day via intravenous injection at dose of 1 mg/kg. The animals were randomly divided into five groups, including the model group (4% DSS), treatment groups (KPV, FK506, and NPs), and a healthy group as control. For chronic colitis, mice were fed 2.5% DSS in drinking water for 5 days, and then given 10 days of normal water, followed by three cycles according to this pattern. The grouping for chronic colitis was consistent with that of acute colitis.

### 2.9 Evaluation of colitis treatment efficacy

The colitis-related disease activity index (DAI) was evaluated and recorded daily during the experimental period, including body weight, rectal bleeding and stool consistency (Liu et al., 2020). The colon (from the cecum to the rectum) was harvested and its length was measured after the experiment. The same colon segment, around 1.0 cm, from each group was fixed in 4% paraformaldehyde for immunohistochemical and histological assessment. Other colon tissues were frozen in liquid nitrogen for analyzing the cytokines and related physiological and biochemical responses (ROS, NO, MPO). In addition, serum samples and main organs were collected for evaluating the treatment efficacy and toxicity.

### 2.10 Statistical analysis

All data were expressed as mean ± SD. Three independent experiments were performed for validity, and at least three samples per test were taken for statistical analysis. Statistical analyses were carried out using GraphPad Prism8.0 (GraphPad Software Inc., La Jolla, CA, United States). Quantification of Western blots was performed with Image J. Statistical comparisons were performed by analysis of variance (ANOVA) and *t* -test. Statistical differences were defined as *p < 0.05, **p < 0.01, ***p < 0.001, and ****p < 0.0001; ns means no significant.

## 3 Results and discussion

### 3.1 Characterization of NPs

**FIGURE 1:**
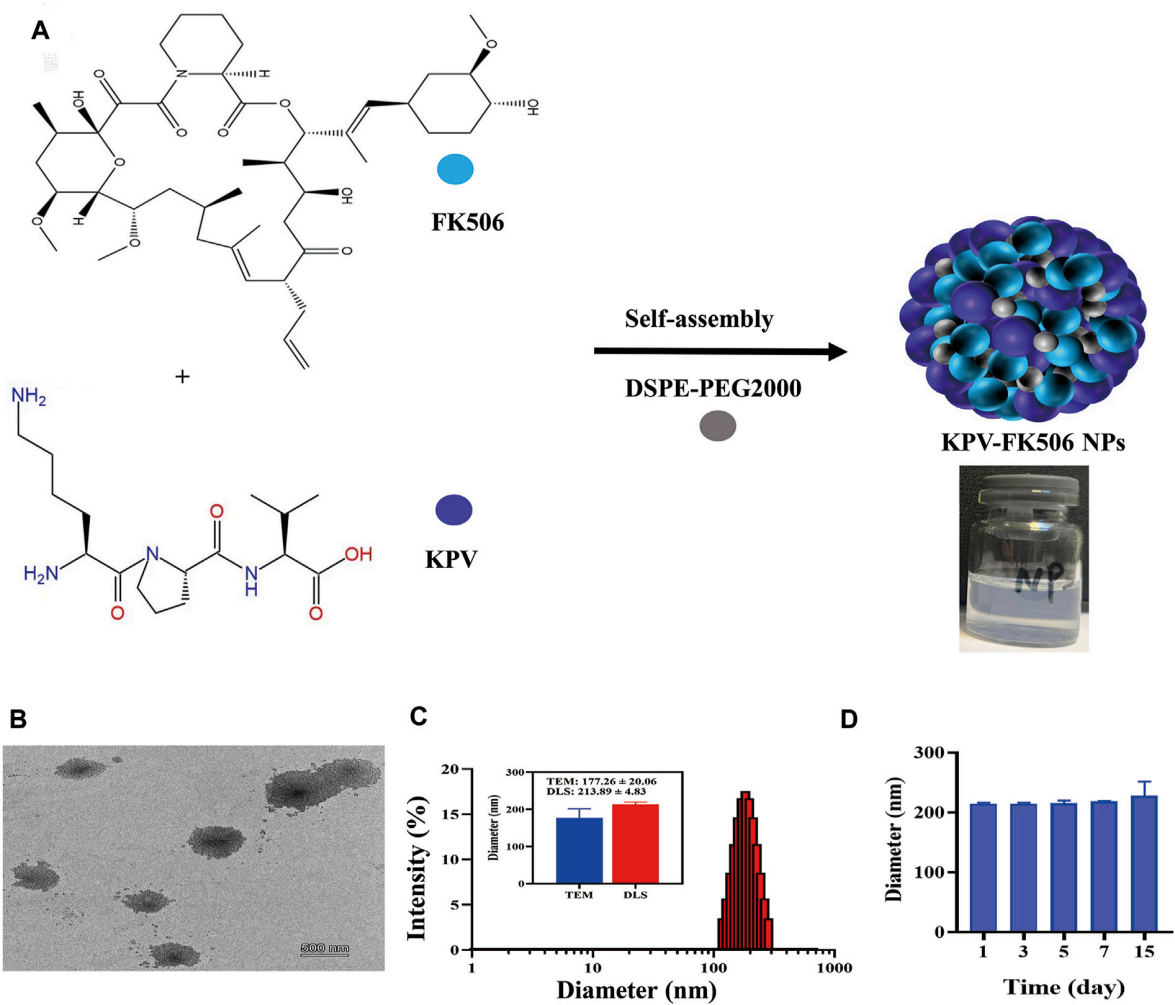
Preparation and characterization of NPs. **(A)** Synthetic scheme of NPs. **(B)**TEM, the scale bars were 500 nm. **(C)** Representative size distribution of NPs. **(D)** The stability of NPs.

The synthesis scheme of **NPs** is shown in Figure 1A, and the picture of NPs presented light blue opalescence (Supplementary Figure S1A). It can be observed that **the NPs** presented spherical morphology with diameters around 177 nm from the representative TEM image (Figure 1B). DLS analysis displayed the hydrodynamic diameter of 214 nm with a polydispersity index of 0.2, and the zeta potential of **NPs** was about −26.7 mV (Figure 1C; Supplementary Figure S1B). Furthermore, the average size of NPs at 1, 3, 5, 7, and 15 days was mostly constant, indicating that the NPs have good stability (Figure 1D). In addition, the results of the hemolysis test revealed the NPs may contribute to reducing the hemolytic effect of FK506 (Supplementary Figure S2). After that, the safety of **NPs** was evaluated by incubating it with macrophages for 24 h, and the results showed that the survival rate of macrophages treated with various concentrations of NPs was greater than 100% (Supplementary Figure S3), suggesting that the NPs did not induce cytotoxicity and can be used for further exploration. Overall, these results indicate that carrier-free NPs with excellent biocompatibility have been successfully synthesized.

### 3.2 PepT1-targeting of NPs

**FIGURE 2:**
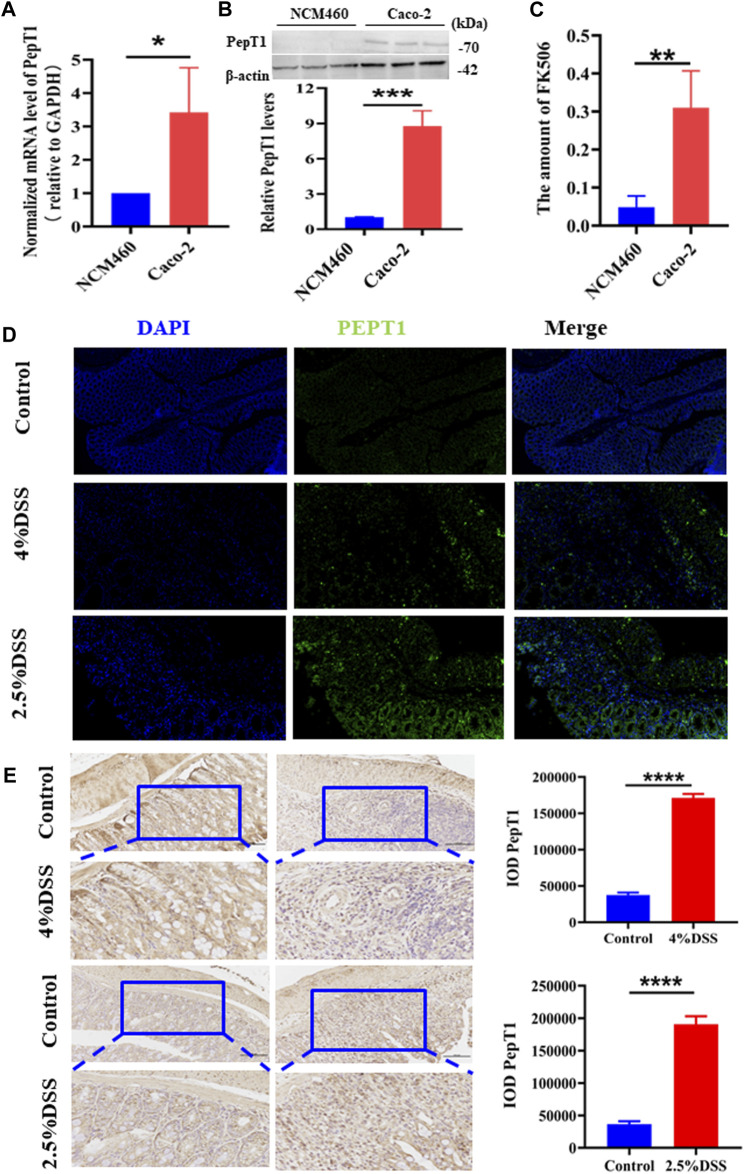
PepT1-targeting of NPs. **(A)** The mRNA expression level of PepT1 in NCM460 and Caco-2. **(B)** The protein expression level of PepT1 in Caco-2 and NCM460. **(C)** The content of FK506 in NCM460 and Caco-2. **(D)** Immunohistochemistry staining of PepT1 in colon tissue of 4% DSS-induced mice and 2.5% DSS-induced mice. The scales at bottom right are 50 μm. **(E)** Immunohistochemical staining of PepT1 in colon tissue of 4% DSS-induced mice and 2.5% DSS-induced mice, the scales at bottom right are 100 μm and 50 μm. Data are expressed as mean SD (n = 3). *p < 0.05, **p < 0.01, ***p < 0.001, ****p < 0.0001; ns, no significance.

NCM460 cells, characterized by low PepT1 expression, and Caco-2 cells, characterized by high PepT1 expression, were utilized to study the targeting efficacy of the NPs. Initially, the expression levels of the PepT1 gene (Figure 2A) and protein (Figure 2B) were confirmed in both NCM460 and Caco-2 cell lines. The obtained results were consistent with the expected outcomes. Subsequently, the intracellular concentration of FK506 was measured by HPLC after the NPs were incubated with the two cell types. As shown in Figure 2C, the amount of FK506 in Caco-2 cells significantly higher than that in NCM460 cells, since the high affinity of KPV to PepT1 allows PepT1 to transport **NPs** into cells with high efficiency (Merlin et al., 2001; Ayyadurai et al., 2013). Studies have revealed that PepT1 is not expressed in normal colonic epithelial cells, while it is abnormally overexpressed in inflammatory cells, such as colonic epithelial cells and macrophages (Charrier et al., 2006; Ayyadurai et al., 2013). Hence, we examined PepT1 expression in the colon through immunohistochemistry staining (IHC) and found that the expression of PepT1 significantly increased in inflamed tissues (Figure 2D). Similarly, colon tissue was inspected using immunohistochemical staining (Figure 2E), and results were consistent with those obtained using IHC in Figure 2D. These results suggest that the NPs could be a potential candidate for the treatment of colitis.

### 3.3 Therapeutic efficacy of NPs in acute colitis

**FIGURE 3:**
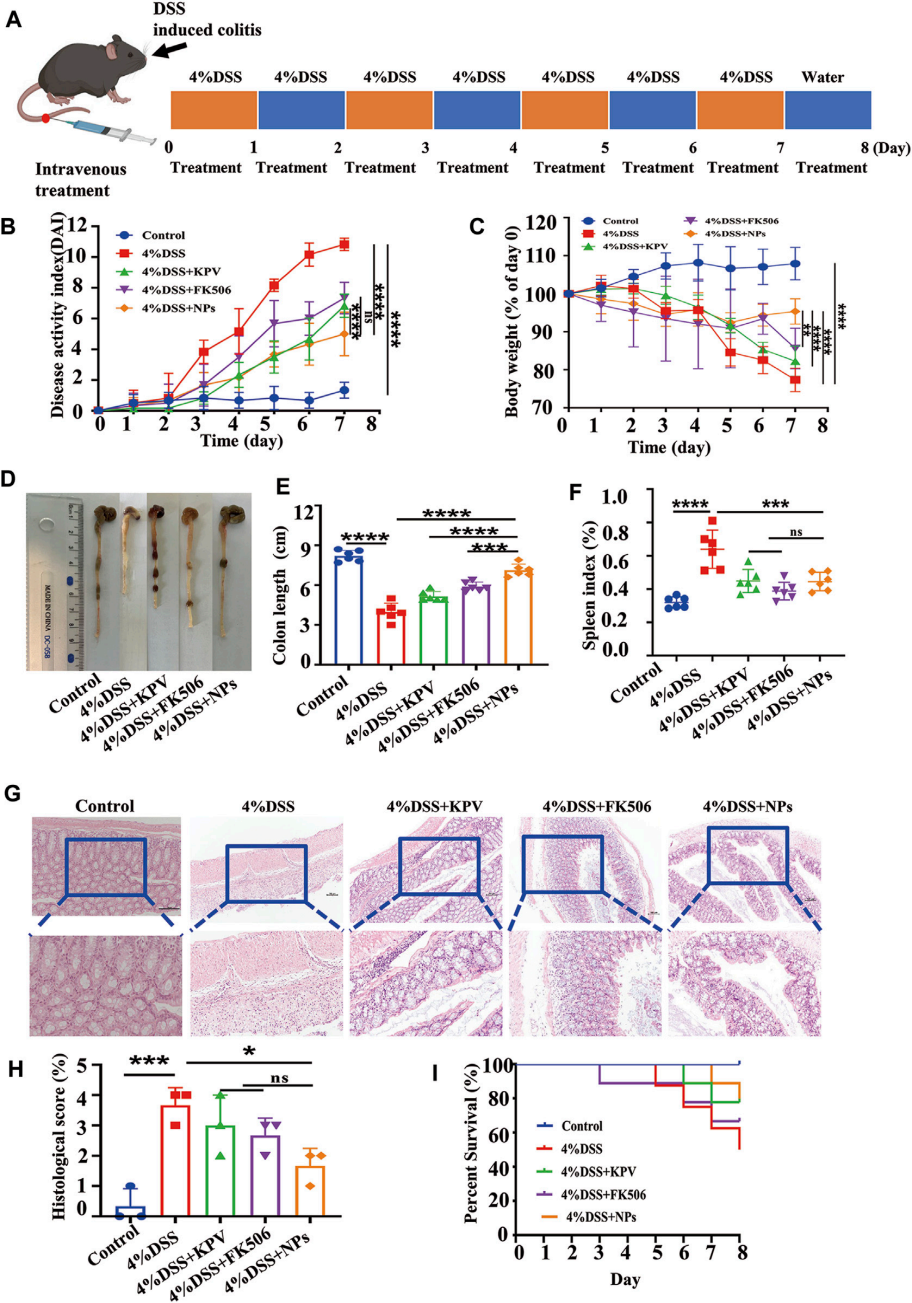
Therapeutic efficacy of NPs in acute colitis. **(A)** C57 mice induced by 4% DSS and the treatment regime with NPs; **(B)** disease activity index (DAI) and **(C)** body weight changes in mice with colitis during the entire treatment period; **(D)** the representative colon graphics and **(E)** the statistics of the colonic length at observation endpoint after various treatments; **(F)** spleen index of mice, represented as a percentage of the spleen weight against each body weight; **(G,H)** Representative H&E-stained sections from the colons in different treatment groups and the histological scores. The scale bars were 100 and 50 μm for the upper and lower panels, respectively; **(I)** survival statistics of mice in different treatment groups. Data are shown as mean ± SD (n = 6). *p < 0.05, **p < 0.01, ***p < 0.001, ****p < 0.0001; ns, no significance.

An acute colitis model was established by providing free drinking water with 4% DSS for 7 days. The mice treated with DSS exhibited symptoms of bloody stool and a positive fecal occult blood test (Supplementary Figures S4A,B). In contrast, normal stool and a relatively clean anus were observed in healthy mice (Supplementary Figures S4C,D). The symptoms exhibited by the mice were similar to those of the mice previously successfully modeled, indicating that we had successfully induced an acute colitis model (Vlantis et al., 2016; Wang et al., 2023). Histological analysis of the main organs revealed no signs of toxicity after 7 days of tail vein injection of NPs, indicating good bio-safety (Supplementary Figures S5, S6). The IBD mice received daily treatment with KPV, FK506, or NPs until sacrifice to evaluate therapeutic efficacy (Figure 3A). In the 4% DSS-induced colitis group, mice exhibited weight loss, increased disease activity index (DAI), and shortened colon length, all of which were significantly improved by treatment with KPV, FK506, or NPs. As shown in Figure 3B, the DAI of the model mice gradually increased with time and eventually reached as high as 11, while the DAI of the NPs group exhibited a significant decline. Weight monitoring results showed that the weight of the inflamed mice recovered after treatment, with the nanoparticle group showing a significant increase in weight compared to the group treated with KPV or FK506 alone (Figure 3C). The length of colon can directly reflect the severity of IBD (Liu et al., 2015). Therefore, we measured the colon length of DSS-treated mice (Figure 3D). As expected, the colon length of colitis mice was significantly shorter than that of healthy mice. The KPV and FK506 treatment groups showed similar mild remission, while the NPs group had a colon length similar to that of the control group (Figure 3E). In addition, DSS treatment led to a significant increase in spleen weight indicating an increase in inflammation levels, and the other treatment groups reversed the results of the DSS treatment group (Figure 3F). Histological analysis of colon tissue revealed that healthy mice had a normal mucosal structure with well-arranged crypts containing plenty of goblet cells, while DSS-induced mice showed mucosal damage, crypt loss, inflammatory cell infiltration, and goblet cell depletion, which is consistent with previous research findings (Ramos and Papadakis, 2019). Furthermore, the HE results of the nanoparticle group’s colon were similar to those of the control group (Figure 3G). What’s more, although there was no difference in the histopathological score between the nanoparticle group and the group treated with KPV or FK506 alone, the score was lowest in the treatment group (Figure 3H). The survival rate of the 4%DSS group was only 60%, whereas the KPV, FK506, and NPs group was 80%, 70%, and 80%, respectively, suggesting the promising potential of NPs for treating colitis (Figure 3I). These results are consistent with the cell-based experiments, indicating that the NPs can effectively treat colitis.

### 3.4 NPs protects against colitis though maintaining intestine barrier

**FIGURE 4:**
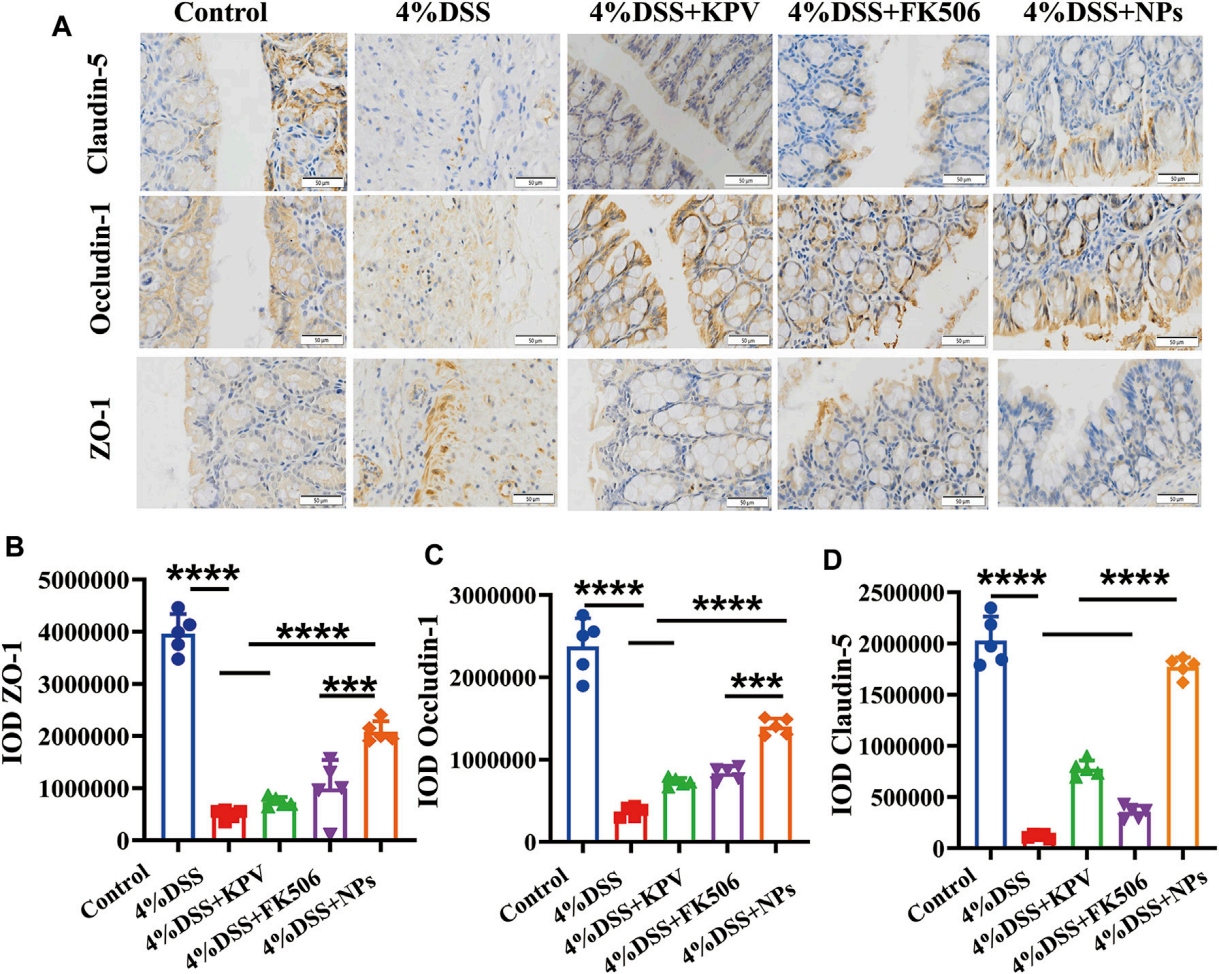
NPs protects against colitis though maintaining intestine barrier. **(A)** The immunohistochemistry staining of Claudin-5, Occludin-1, and ZO-1 in colon tissues of 4% DSS. The quantification of **(B–D)**. The scales at bottom right are 50 μm *p < 0.05, **p < 0.01, ***p < 0.001; ns, no significance.

The tight junctions between the cells of the intestinal epithelium, which are made up of ZO-1, claudin-1, and Occludin-1g proteins, are essential for maintaining the barrier function of the intestinal cells (McLeod et al., 2019; L'Huillier et al., 2019). To explore the influence of NPs on the integrity of the intestinal epithelial barrier, intestinal tight junctions were studied using IHC. The protein expression levels of ZO-1, claudin-1, and Occludin-1g were considerably reduced in colon tissue of the DSS-treated group (Figure 4). The expression of KPV and FK506 was mildly reduced in the treatment group, whereas it was relatively unaffected in the NPs treatment group, and contributed to alleviate DSS-induced colonic inflammation.

### 3.5 NPs protect against colitis though reducing inflammation

Research has found macrophages play a critical role in the pathogenesis of colitis (Varol et al., 2009; Zigmond et al., 2012; Asano et al., 2015; Li et al., 2017). Based on this, RAW264.7 cells were applied to investigate the anti-inflammatory activity of NPs *in vitro*. Firstly, the optimal intervention concentration and timing were determined by measuring the levels of inflammatory cytokines in LPS-stimulated macrophages after treatment with NPs. As shown in Supplementary Figure S7, the mRNA levels of IL-6, IL-1β, and TNF-α were significantly decreased when NPs were administered at a concentration of 10 μg/mL for 6 h. And this dose regimen was used in the subsequent experiments. The mRNA expression of inflammatory indicators (TNF-α, IL-1β, IL-6, IL-2) was markedly increased in the LPS group and notably decreased in the therapeutic group (Supplementary Figures S8A–D). Notably, FK506 prevents T cell activation by inhibiting interleukin-2. Besides, it can suppress the proliferation of cytotoxic T cells and the expression of interleukin-2 receptors (Bitton et al., 2012). Therefore, we further detected IL-2 protein levels through Western blot, with statistically significant results shown in Supplementary Figures S8E,F. Moreover, results showed NPs significantly reduced the mRNA expression of TNF-α, compared with free KPV and FK506 (Supplementary Figure S8A). The findings are in line with existing literature, Xiao et al. found that LPS-treated Raw 264.7 macrophages pre-treated with NPs could significantly decreased the levels of TNF-α (Xiao et al., 2017). Wang et al. also reported a reduction in TNF-α levels after FK506 nanoparticles were administered (Xiao et al., 2017). Overall, these studies suggest that further studies are needed to explore the potential for treating colitis *in vivo* using NPs.

**FIGURE 5:**
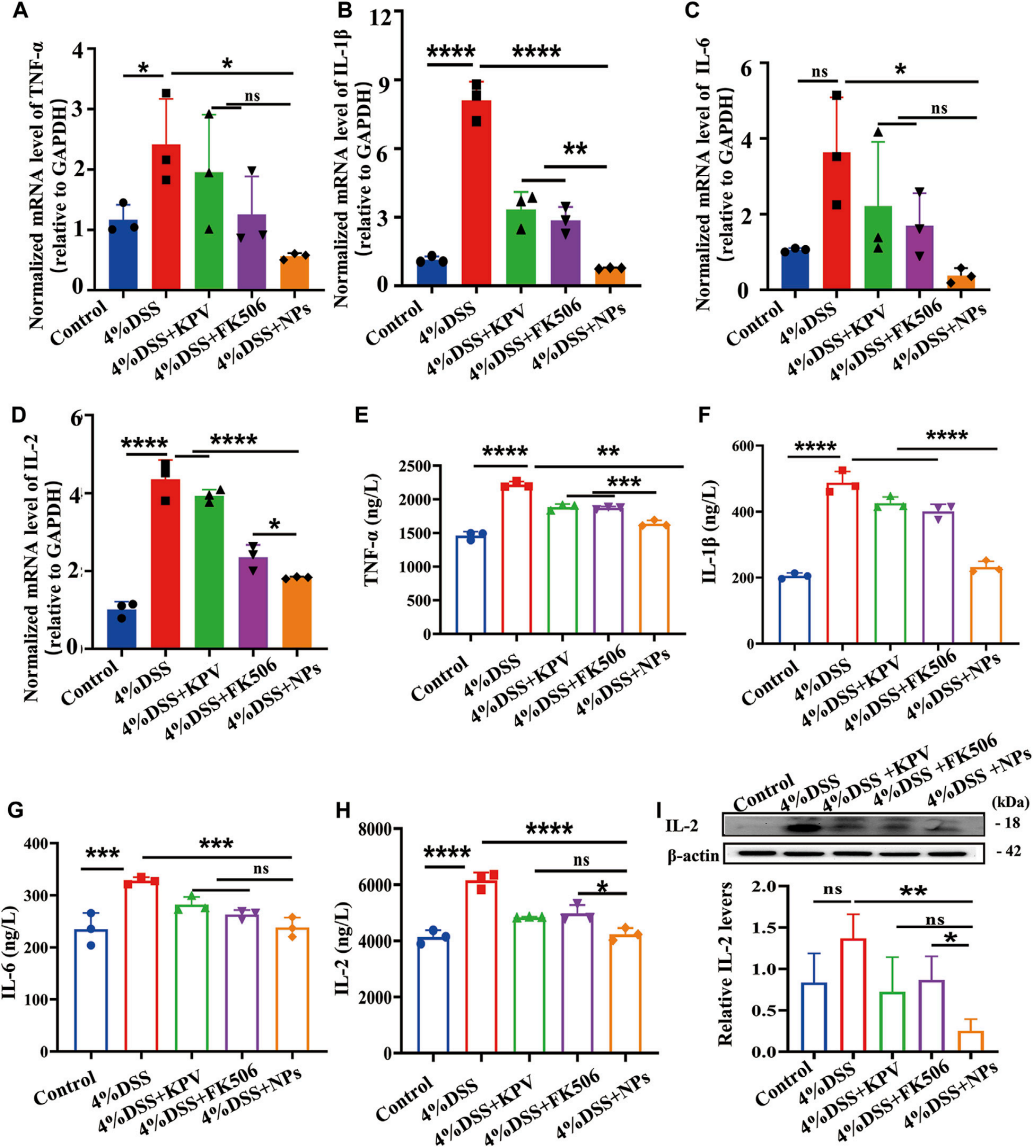
NPs protect against colitis though reducing inflammation. **(A)** TNF-α, **(B)** IL-1β, **(C)** IL-6, **(D)** IL-2 in the colons detected by qPCR for 4% DSS, **(E)** TNF-α, **(F)** IL-1β, **(G)**IL-6, **(H)** IL-2 in the colons detected by ELISA. **(I)** Protein expression level of IL-2 examined by WB. Data are expressed as mean SD (n = 3). *p < 0.05, **p < 0.01, ***p < 0.001; ns, no significance.

Hereby, the mRNA and protein expression level of pro-inflammation cytokines (TNF-α, IL-1β, IL-6, IL-2) were detected by real-time PCR and ELISA after treatment of colitis. As shown in Figures 5A–D, the colitis group showed the highest mRNA expression of TNF-α, IL-1β, IL-6, IL-2, and the mRNA expression of these pro-inflammation cytokines were decreased by KPV and FK506. As expected, NPs showed the best treatment effect. At the protein level, results represented that pro-inflammatory cytokines were reduced in the blood serum for all treated mice compared to the colitis group, with NPs exhibiting the most pronounced effects (Figures 5E–H). Results of the Western blot showed IL-2 protein levels significantly decreased after NPs treatment (Figure 5I), consistent with above results.

### 3.6 NPs against inflammation though decreasing immune cell infiltration

**FIGURE 6:**
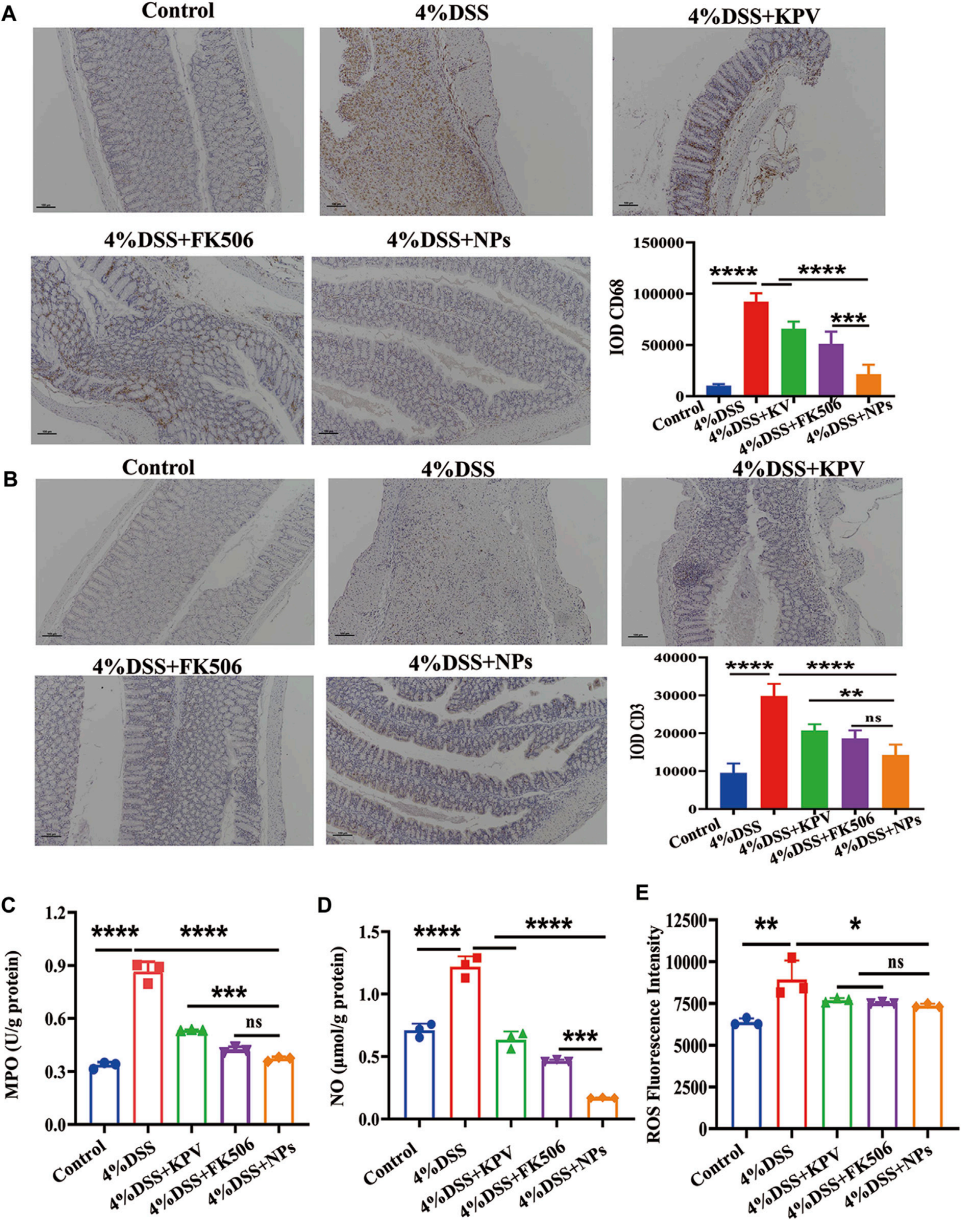
NPs against inflammation though decreasing immune cell infiltration. **(A)** CD68, **(B)** CD3 in the colons detected by IHC for 4% DSS, the scales at bottom right are 100 μm. **(C)** MPO, **(D)** NO, **(E)** ROS in the colons detected by ELISA. Data are expressed as mean SD (n = 3). *p < 0.05, **p < 0.01, ***p < 0.001; ns, no significance.

Macrophages and T lymphocyte are involved in the immune processes of IBD, which secrete a large number of inflammatory factors when activated (Khor et al., 2011; Nakase et al., 2022). In addition, FK506, as an immunosuppressive agent, can inhibit the proliferation and activation of immune cells. Therefore, we hypothesized that NPs might reduce inflammation by reducing macrophage and T lymphocyte infiltration. Then, we employed IHC to assess the expression of CD68 and CD3, since CD68 and CD3 are macrophage and T lymphocyte surface markers, respectively (Sosman et al., 2008; Lei and Fan, 2022). The expression of CD68 and CD3 was significantly elevated in the 4% DSS group, while the NPs group showed significantly lower expression levels than both the KPV and FK506 groups (Figures 6A,B). Myeloperoxidase (MPO), a biomarker of neutrophil filtration, plays a crucial role in the development of UC (Masoodi et al., 2011; Garrity-Park et al., 2012; Xiao et al., 2014). It increased significantly in inflammatory colon tissues (Figure 6C), indicating that a large number of neutrophils infiltrated the colon. And the NPs dramatically decreased the MPO level, with much more pronounced effects than KPV and FK506. Similarly, NO and ROS in the NPs treated group were also the lowest compared to other colitis groups (Figures 6D,E).

### 3.7 Therapeutic efficacy of NPs in chronic colitis

**FIGURE 7:**
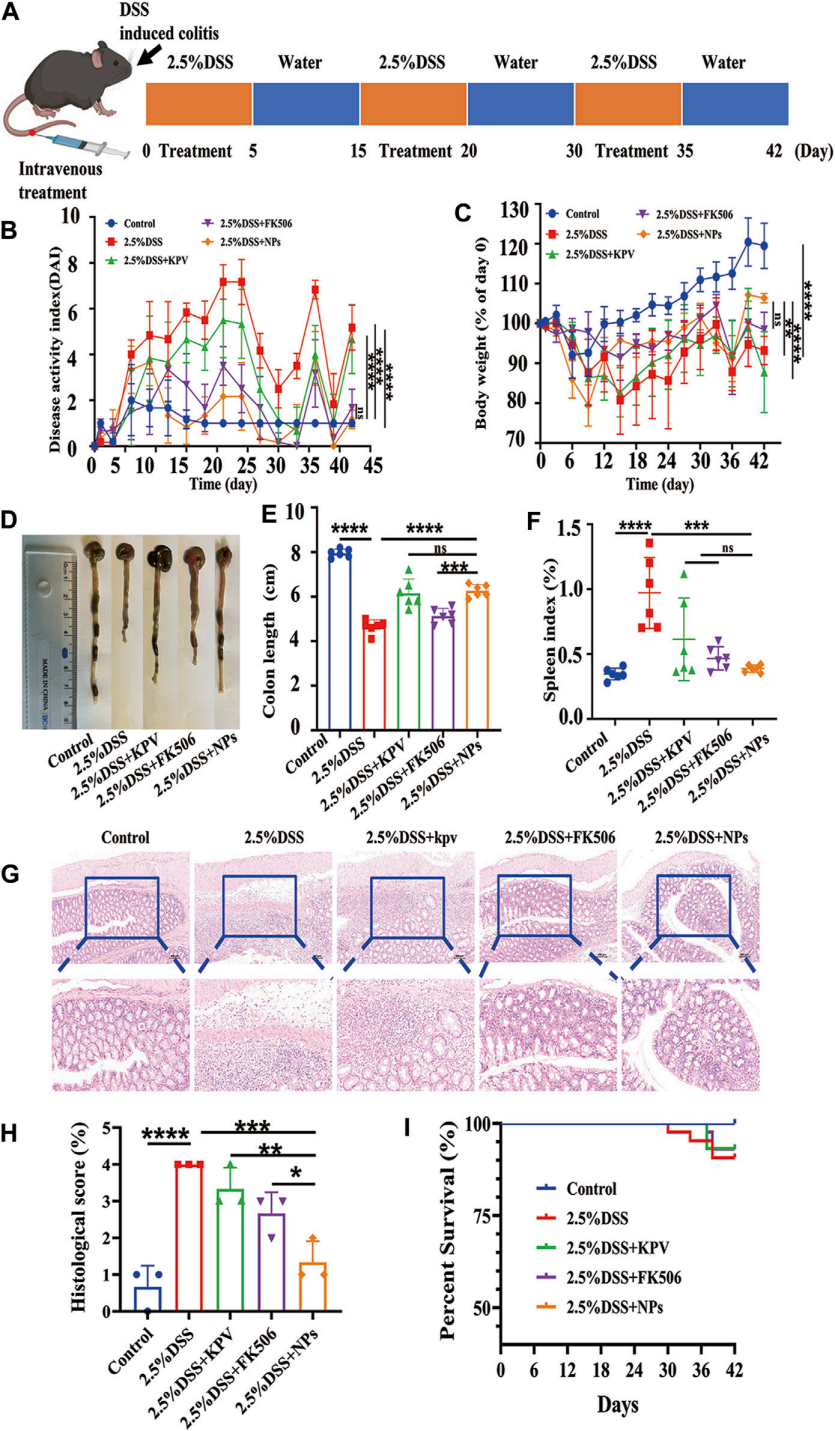
Therapeutic efficacy of NPs in chronic colitis. **(A)** C57 induced by 2.5%DSS and the treatment regime with NPs; **(B)** disease activity index (DAI) and **(C)** body weight changes for mouse with colitis during the entire treatment period; **(D)** the representative colon graphics and **(E)** the statistics of the colonic length at observation endpoint after various treatments; **(F)** spleen index of mice, represented as a percentage of the spleen weight against each body weight; **(G,H)** Representative H&E-stained sections from the colons in different treatment groups and the histological scores. The scale bars were 100 μm and 50 μm for the upper and lower panel respectively; **(I)** Survival statistics of mice in different treatment groups. Data are shown as mean ± SD (n = 6). *p < 0.05, **p < 0.01, ***p < 0.001, ****p < 0.0001; ns, no significance.

As chronic inflammation exhibits PepT1 overexpression more than acute inflammation, we investigated the role of NPs in 2.5% DSS-induced chronic colitis. Following a modified protocol, mice with chronic colitis were induced with 2.5% DSS through oral gavage for 5 days, while being concurrently administered the drug via tail vein injection, followed by free access to drinking water for 10 days for three cycles (Figure 7A). Comparable doses of FK506 and NPs were administered during acute and chronic inflammation. Mice treated with 2.5% DSS displayed obvious blood in their stool and positive fecal occult blood test (Supplementary Figures S9A,B), while healthy mice displayed normal stool and relatively clean anus (Supplementary Figures S9C,D). The safety of NPs was assessed using histological analysis of the main organs, which showed no indications of toxicity after three cycles of tail vein administration (Supplementary Figures S10, S11). Our preliminary data indicate that NPs exhibit good biocompatibility and are suitable for *in vivo* application. After three treatment cycles, the DAI score, weight loss, colon length, spleen index, H&E staining, and survival rate were used to evaluate the severity of mouse colitis and the therapeutic effect of NPs.

The results showed that the DAI score of mice with colitis in 2.5% DSS group was higher than that in other colitis treatment groups, whereas the NPs and healthy groups had similar scores (Figure 7B). In terms of weight loss, the 2.5% DSS group was close to the KPV treatment group (Figure 7C), which may be related to individual differences in autoimmunity among the mice. The colon length of the 2.5% DSS group was significantly shorter than that of the other treatment groups, with the NPs group having the second-longest colon length after the healthy group (Figures 7D,E). In addition, the spleen index in the KPV, FK506, and NPs groups was lower than that in the 2.5% DSS group (Figure 7F). After that, H&E staining was used to examine the colon tissue (Figure 7G). The colon tissue of the control and NPs groups had a typical mucosal shape with conspicuous, orderly crypts that were densely packed with goblet cells. DSS-induced mice and KPV-treated mice, on the other hand, exhibited mucosal damage and typical pathological traits, such as crypt loss, inflammatory cell infiltration, and goblet cell depletion. After treatment of FK506, the colon tissue showed remarkable recovery, showing only infiltration of inflammatory cells. For histopathological score, the model group exhibited the highest score of 4 due to its more severe condition, while scores decreased in all treatment groups. The nanoparticle group had the most significant decrease, and there was a statistically significant difference between this group and the other treatment groups (Figure 7H). The mice exposed to 2.5% DSS had a survival rate of 70% in Figure 7I, while KPV and FK506 both reduced the death rate of these mice by 10%. Notably, all colitis mice in the NPs group survived.

### 3.8 NPs protect against chronic colitis though reducing inflammation and maintaining intestine barrier

**FIGURE 8:**
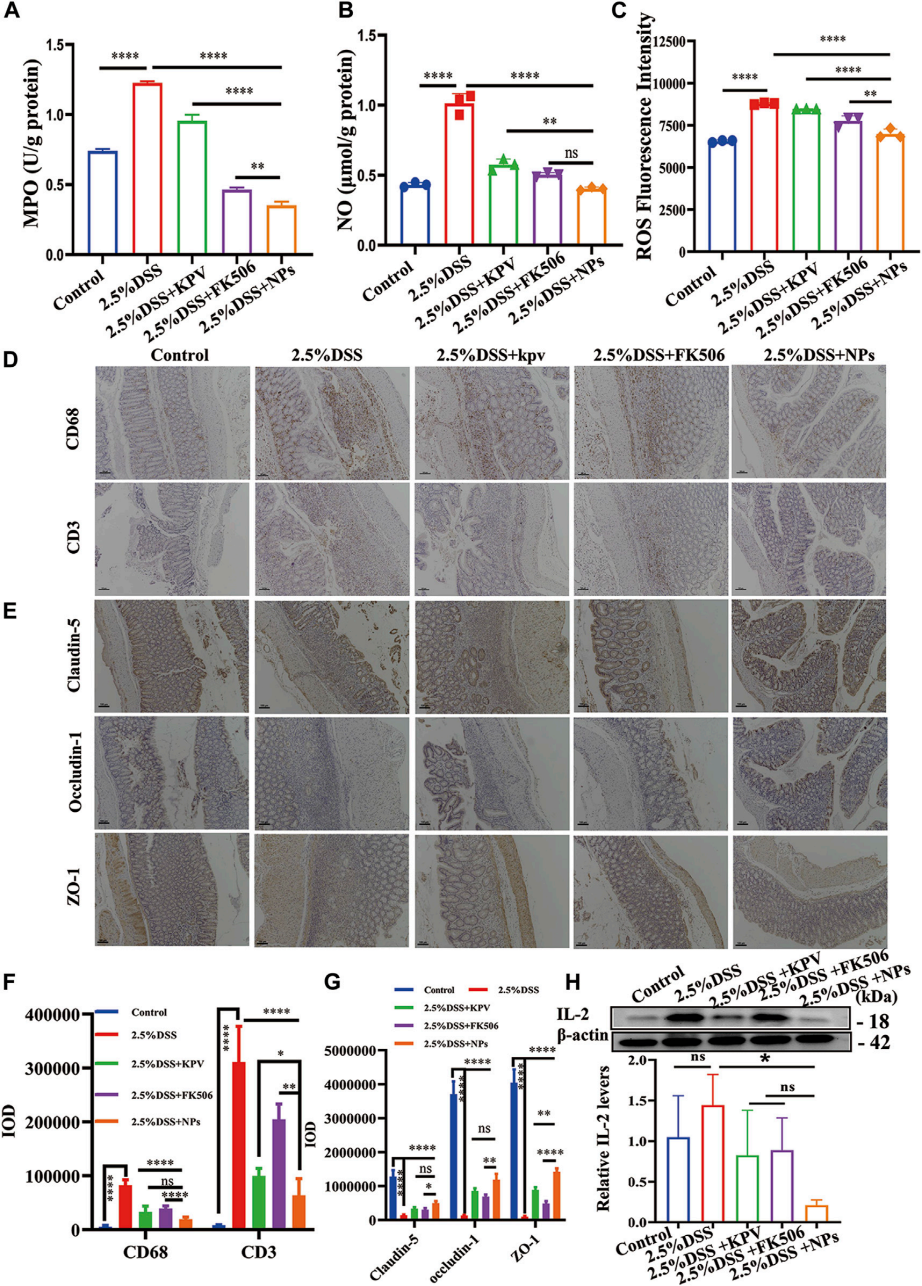
NPs protect against chronic colitis by reducing inflammation and maintaining the intestine barrier. **(A)** MPO, **(B)** NO and **(C)** ROS activity in 2.5% DSS colon tissues in different treatment groups (n = 3). **(D)** The immunohistochemistry staining of CD68 and CD3; the scales at the bottom right are 100 μm. **(E)** The immunohistochemistry staining of tight junction proteins (Claudin-5, Occludin-1, and ZO-1) in colon tissues of 2.5% DSS; the scales at the bottom right are 100 μm. **(F,G)** The quantification of **(D,E)**. **(H)** protein expression level of IL-2 examined by WB. Data are expressed as mean SD (n = 3). *p < 0.05, **p < 0.01, ***p < 0.001, ****p < 0.0001; ns, no significance.

The increased content of MPO, NO, and ROS in colon tissue provided further evidence of the significant inflammatory reaction induced by 2.5% DSS in mice. It can be observed that these indicators significantly decrease after treatment, with the NPs group showing the most significant improvement. NPs might have a more substantial impact on MPO and ROS since both demonstrated statistically significant differences compared to FK506 alone, which showed no significance in acute colitis (Figures 8A–C). In addition, closely linked proteins play an indispensable role in normal colon tissue. In order to further verify the therapeutic effect of NPs in chronic colitis induced by 2.5% DSS, immunohistochemical staining of CD68, CD3, Claudin-5, Occludin-1, and ZO-1 was measured in colon tissue. Results showed that CD68 and CD3 expression significantly increased in the 2.5% DSS group, while the NPs group expression was significantly lower than that of the KPV and FK506 groups. This suggests that macrophages and T cells in the 2.5% DSS group are activated, while macrophages and T cells in other treatment groups, especially in the NPs group, are reduced and similar to those in the healthy group (Figures 8D,F). Besides, immunohistochemical results showed that the expression of Claudin-5, Occludin-1, and ZO-1 in the colon tissue of 2.5% DSS group mice was significantly lower than that of the healthy group. The NPs group was second only to the healthy group, and other treatment groups were between 2.5% DSS group and NPs group (Figures 8E,G). At the same time, we also detected IL-2 in colon tissue. The expression of this protein was significantly higher in the control group. The KPV group and FK506 groups were lower than the 2.5% DSS inflammation group, while the inflammation in the NPs group was significantly lower than in other treatment groups (Figure 8H). It is widely recognized that macrophages are crucial therapeutic targets in the treatment of IBD (Bitton et al., 2012). Our study demonstrates that self-assembled NPs can reduce inflammation and maintaining intestine barrier in acute and chronic colitis induced by DSS.

Additionally, previous studies indicated that tumor necrosis factor was a major therapeutic target in IBD (Liu et al., 2020), In animal models of IBD, the inhibition of inducible NO synthase (iNOS) could reduce the severity of the IBD and epithelium apoptosis, TNF-α enhances antimicrobial capabilities and contributes to macrophage activation as well (Podolsky, 2002). Moreover, it has confirmed the effect of FK-506 can significantly decreased TNF-a, IL-1β, and IL-6 levels, inhibiting the activation of NF-κB (Liu et al., 2017). ELISA was used to detect the serum TNF-α of each group of mice (Supplementary Figure S12A), and the results showed that the TNF-α of the NPs group was lower than that of the other treatment groups, which may be macrophages produce more reactive nitrosative species (RONS) in response to TNF-α, including nitric oxide (NO−) and its metabolite, peroxynitrite (ONOO−) (Parameswaran and Patial, 2010). IL-1β is an important role of the pathogenesis of IBD (Tye et al., 2018), a certain quantity of IL-1β is involved in repair of intestinal epithelial cells and reconstitution of the epithelial barrier during the resolution of colitis, but its deficiency or over-expression could correlate with disease exacerbation (Bersudsky et al., 2014). IL-1β is increased in the mucosa of patients with IBD and was associated with the proliferation of pathogenic T helper 17 (Th17) cells (Eastaff-Leung et al., 2010). In our study, the serum IL-1β of each mouse group was measured by using ELISA, and the findings revealed that the NPs group’s IL-1β was lower than the other treatment groups (Supplementary Figure S12B), which might indicate the newly co-assembled of NPs might have potential to significantly less gut inflammation and damage to the intestinal permeability though relieving the IL-1β express. Besides TNF-α and IL-1β, the best characterized inflammatory cytokine is IL-6, which also plays a significant role in the pathogenesis of IBD (Gerlach et al., 2012). Although the high levels of IL-6 can weaken the body’s anti-inflammatory immunity, the presence of FK506 has reduced levels of the serum inflammatory cytokines in functionally impaired virus-specific T cells generated (Araki et al., 2010). ELISA was used to test the blood IL-6 of each mouse group at our study (Supplementary Figure S12C), the results showed that the NPs group had lower IL-6 than the treatment of FK506 groups and KPV groups, which implied that treatment of NPs was involved in the chronic phases of experimental colitis by reduce the expression of IL-6. According to recent lines of evidence, IBD patients have reported releasing IL-2 as a result of such events (Clos-Garcia et al., 2019). The pathophysiology of intestinal inflammation is influenced by an abnormal balance between Tregs and Teff, a great amount of data has supported that the IL-2/Treg axis represents a possible therapeutic target for IBD (Abo et al., 2019). In a recent trial, FK506 has been demonstrated in restraining the IL-2 expression (Wu et al., 2019b). In order to further confirm the interaction between NPs and IL-2, the level of IL-2 was tested using ELISA in each mouse group’s blood (Supplementary Figure S12D). The results revealed that the NPs group had lower IL-2 than the other treatment groups, which indicated that the NPs might inhibit the IL-2 express to promote T cell proliferation and differentiation to relieve the inflammation in IBD. Next, we examined TNF-α, IL-1β, IL-2, IL-6 and IFN-γ mRNA expression in mouse colon tissue by qPCR (Supplementary Figure S13). The mRNA results revealed that the treatment effect of the NPs groups was significantly better than that of other treatment groups, were consistent with those of mouse serum ELISA and previous studies. Those results showed that the NPs significantly inhibited the TNF-a, IL-1b, and IL-6 levels to relieve the inflammation in IBD.

The majority of current IBD research focuses solely on acute or chronic cases. We have prepared a comparison between acute and chronic studies, providing a nanomedicine that can treat both acute and chronic inflammation. And the self-assembly of KPV on FK506 for functionalized carrier free self-assembly is the first of its kind. The effectiveness of KPV by itself is inferior to that of NPs, regardless of the kind of enteritis: acute or chronic. This could be because KPV has a short half-life and is easily broken down (Tang et al., 2004; Shoombuatong et al., 2018), which makes it more stable when made into NPs. In addition, the results of this study acute and chronic inflammation reveal that NPs have a safe and effective therapeutic effect on colitis, regardless of the type of inflammation. This could be explained by the synergistic effect of KPV and FK506. On the other hand, NPs can be transported to cells through PepT1, which is related to the high affinity between KPV and PepT1 and the specific expression of inflammatory cell transporters, indicating that the NPs can be collected in a colon that is inflamed. Due to the high drug loading capacity and ability to avoid issues such as complex processing and systemic metabolism associated with carrier materials, carrier-free NPs have strong potential for clinical translation (Huang et al., 2021; Karaosmanoglu et al., 2021). There are still some limitations in this paper. First, the long-term toxic effects of nanomaterials on enteritis. Although the toxic effects of acute and chronic enteritis were preliminatively evaluated in this paper, the maximum use cycle of animals is only 2 months. Enteritis is a process of repeated attacks and medication, and the effects of long-term repeated medication on the body need to be tested. The second is the effect of nanomaterials on acute and chronic enteritis which is more effective, these are the next step for in-depth discussion.

## 4 Conclusion

As a novel nanodrug with PepT1-targeting capacity, the self-assembled NPs significantly alleviated murine acute and chronic colitis symptoms. And NPs can preserve the integrity of the epithelial barrier by attenuating the loss of tight junction proteins, such as ZO-1 and Occludin-1 proteins. Additionally, the NPs treatment decreases inflammation by reducing the infiltration of macrophages and T lymphocytes, resulting in the downregulation of inflammatory cytokines, ROS, NO, and MPO. In conclusion, NPs exhibit a unique role in relieving acute and chronic colitis and possess potential application ability for treating immune-related inflammatory diseases.

## Data Availability

The original contributions presented in the study are included in the article/Supplementary Material, further inquiries can be directed to the corresponding authors.
