# Supplementary material for: PepT1-targeted nanodrug based on co-assembly of anti-inflammatory peptide and immunosuppressant for combined treatment of acute and chronic DSS-induced ColitiS
Source: Front Pharmacol. 2024 Aug 15;15:1442876. doi: 10.3389/fphar.2024.1442876 (PMC11357942; doi:10.3389/fphar.2024.1442876)
Supplement: Supplementary file 1 [file DataSheet1.PDF]

Supplementary Material

PepT1-targeted Nanodrug Based on Co-Assembly of Anti-inflammatory Peptide and Immunosuppressant for Combined Treatment of Acute and Chronic DSS-induced Colitis

Daifang Zhang# [1,3], Longqi Jiang# [2,3], Fengxu Yu [1,3], Pijun Yan [3,5], Yong Liu [2,3,4]*, Ya Wu [2,3,4]*, Xi Yang [2,4]*

[1]Department of Cardiovascular Surgery, The Affiliated Hospital of Southwest Medical University, 646000 Luzhou, China.

[2]Department of Vascular Surgery, The Affiliated Hospital of Southwest Medical University, 646000 Luzhou, China.

[3]Metabolic Vascular Disease Key Laboratory of Sichuan Province, The Affiliated Hospital of Southwest Medical University, 646000 Luzhou, China.

[4]Key Laboratory of Medical Electrophysiology, Ministry of Education & Medical Electrophysiological Key Laboratory of Sichuan Province (Collaborative Innovation Center for Prevention of Cardiovascular Diseases), Institute of Cardiovascular Research, Southwest Medical University, Luzhou 646000, China.

[5]Department of Endocrinology and Metabolism, The Affiliated Hospital of Southwest Medical University, Luzhou, 646000, Sichuan, China.

*****Corresponding author.

#Contributed equally.

*****Correspondence: Yong Liu, lyong74@163.com; Ya Wu, yawu1993@163.com; Xi Yang, Xiyang2024520@163.com

[†] These authors have contributed equally to this work.

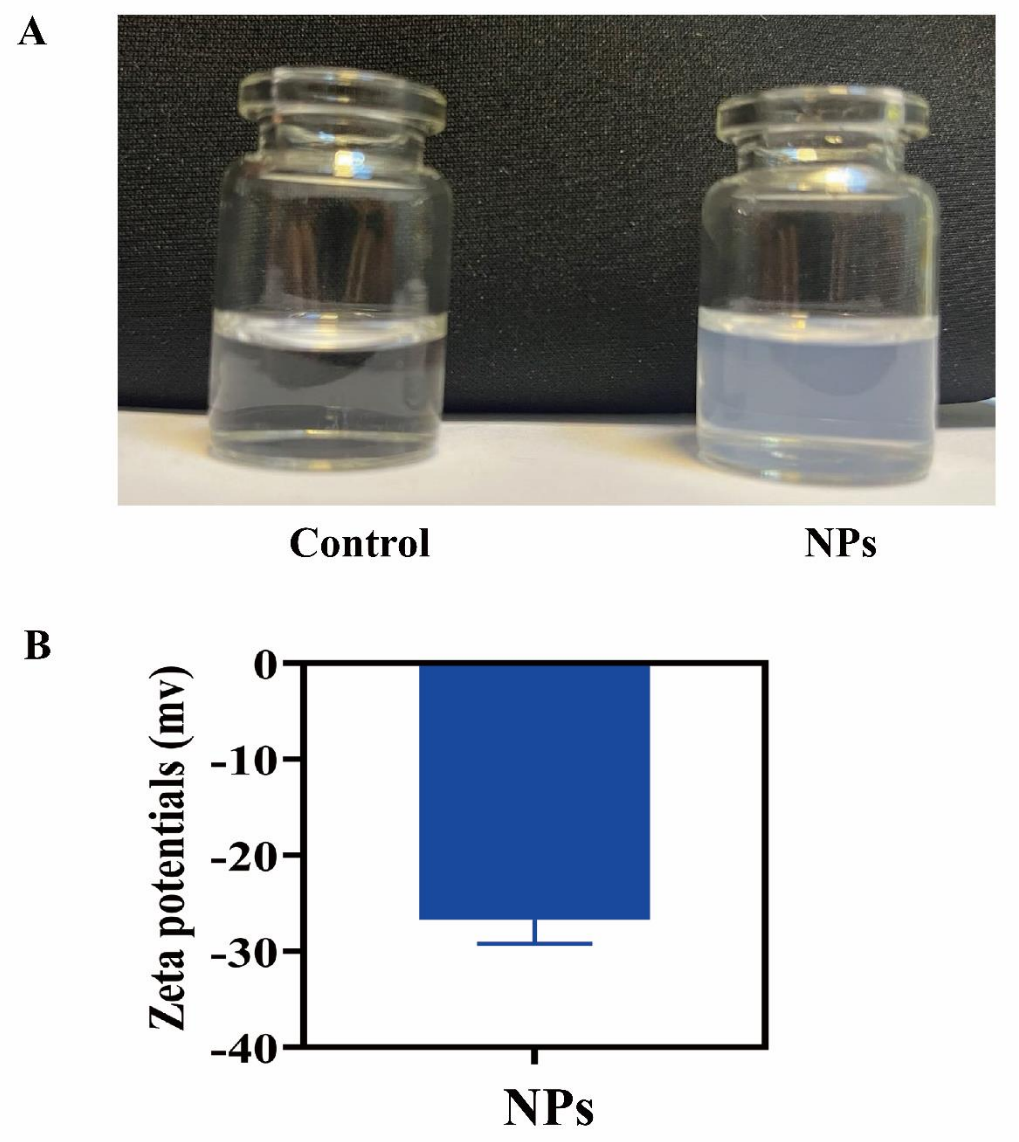

Fig S1. A. Image of NPs nanoparticles; B. Zeta potential of NPs

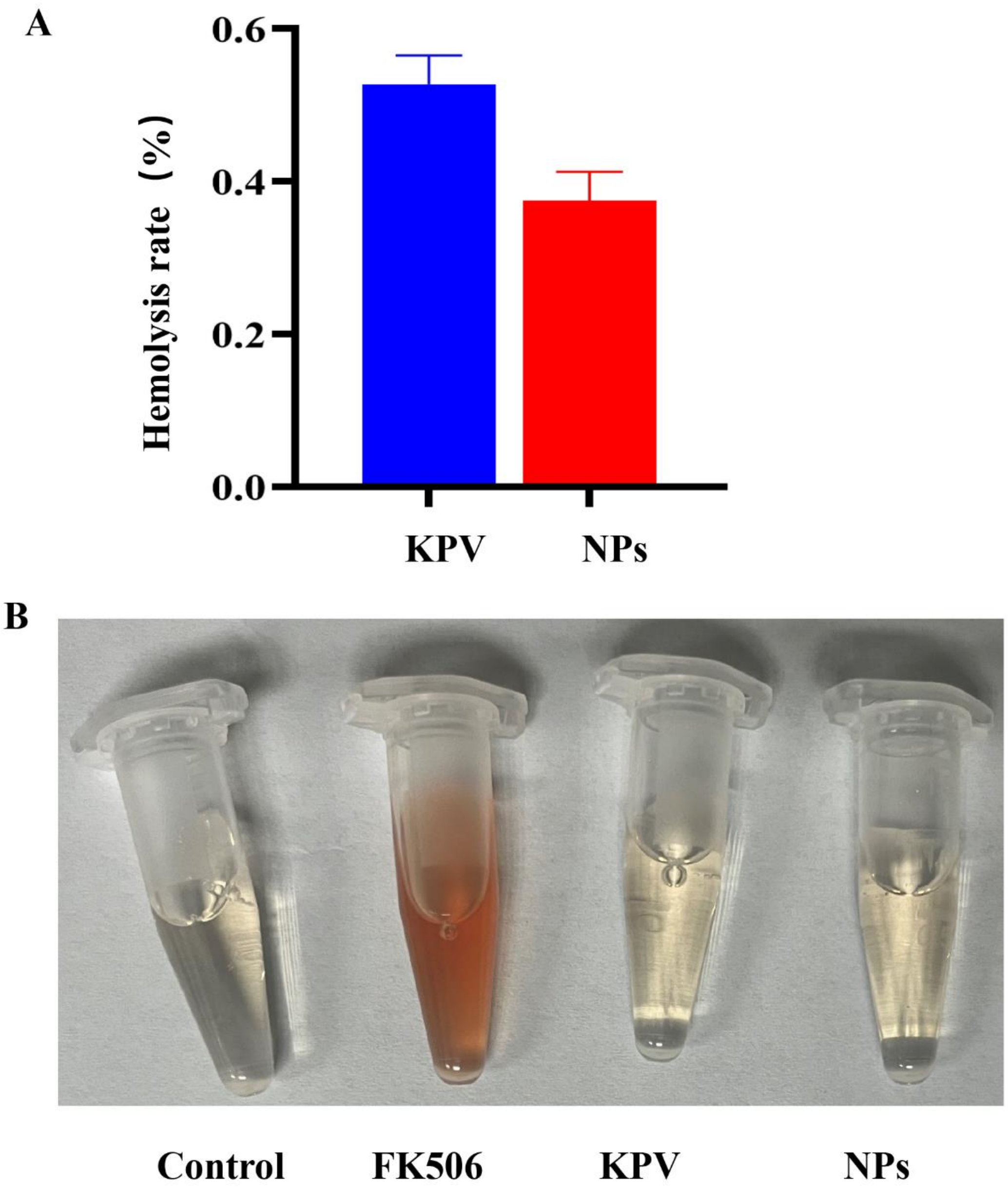

Fig S2. A. Hemolysis rate of KPV and NPs; B. Hemolysis comparison of each group.

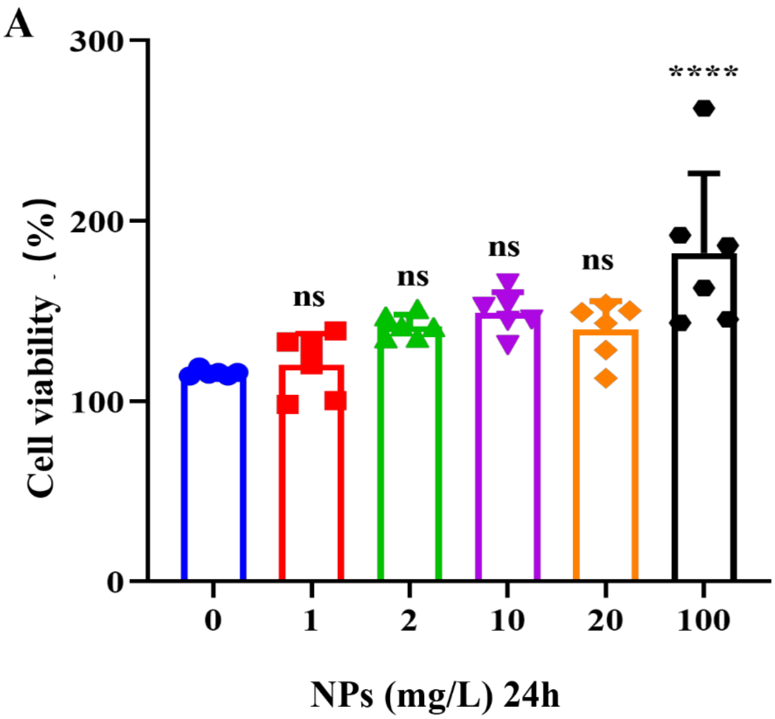

Fig S3. A. Cell survival rate of NPs at 24 hours, **P* < 0.05, ***P* < 0.01, ****P* < 0.001, *****P* < 0.0001; ns, no significance.

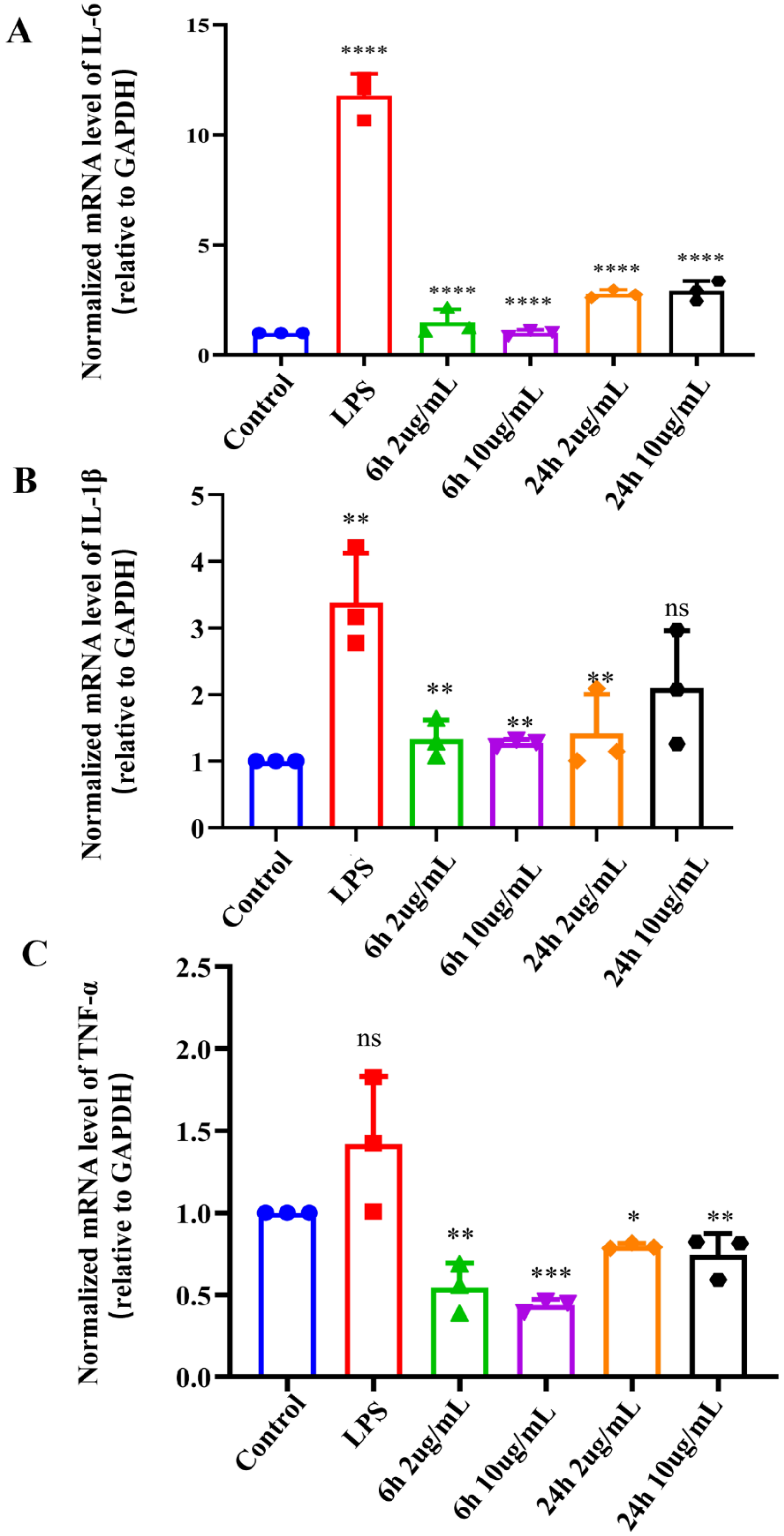

Fig S4. NPs concentration and time mRNA level of (A)IL-6, (B)IL-1β, (C) TNF-α at different NPs concentrations and times, *$P < 0.05$, **$P < 0.01$, ***$P < 0.001$, ****$P < 0.0001$; ns, no significance.

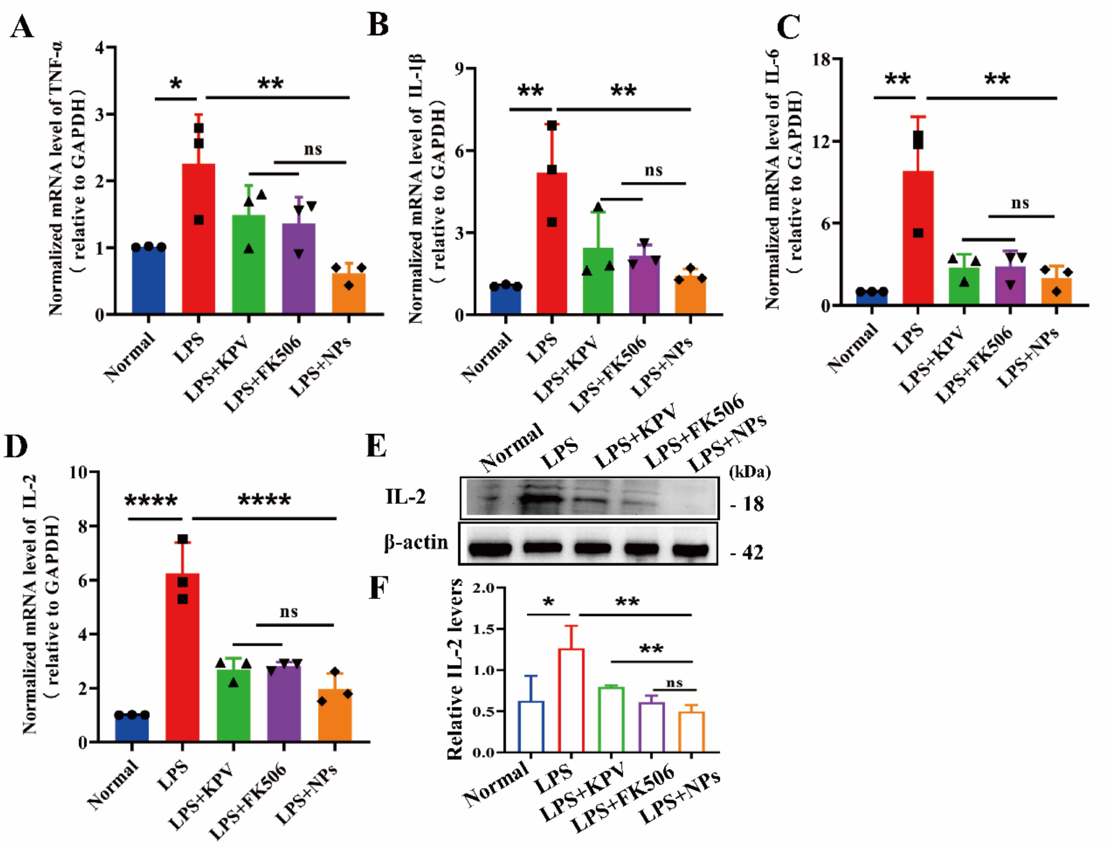

Fig S5. The mRNA expression of inflammatory indicators level of (A) TNF-α, (B)IL-1β, (C) IL-6, (D) IL-2 in LPS administration, (E) and (F) The protein expression level of IL-2 in Raw264.7. *P < 0.05, **P < 0.01, ***P < 0.001, ****P < 0.0001; ns, no significance.

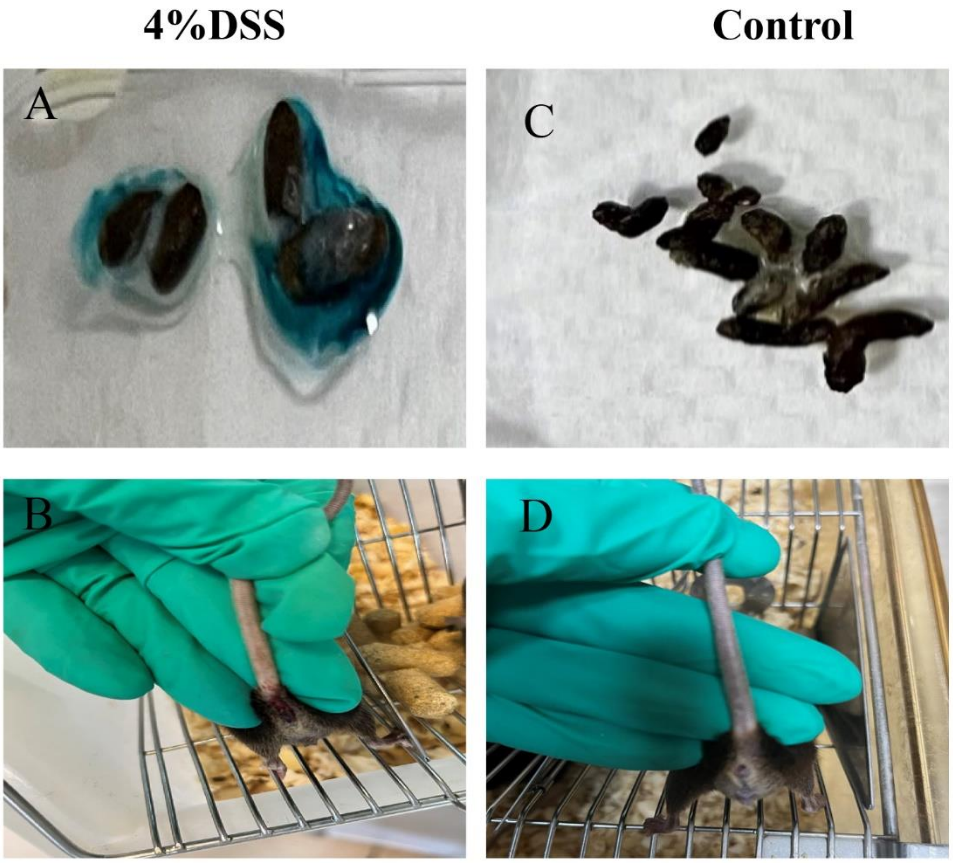

Fig S6. 4%DSS experimental group (A), (B) and Occult blood test in control group(C), (D).

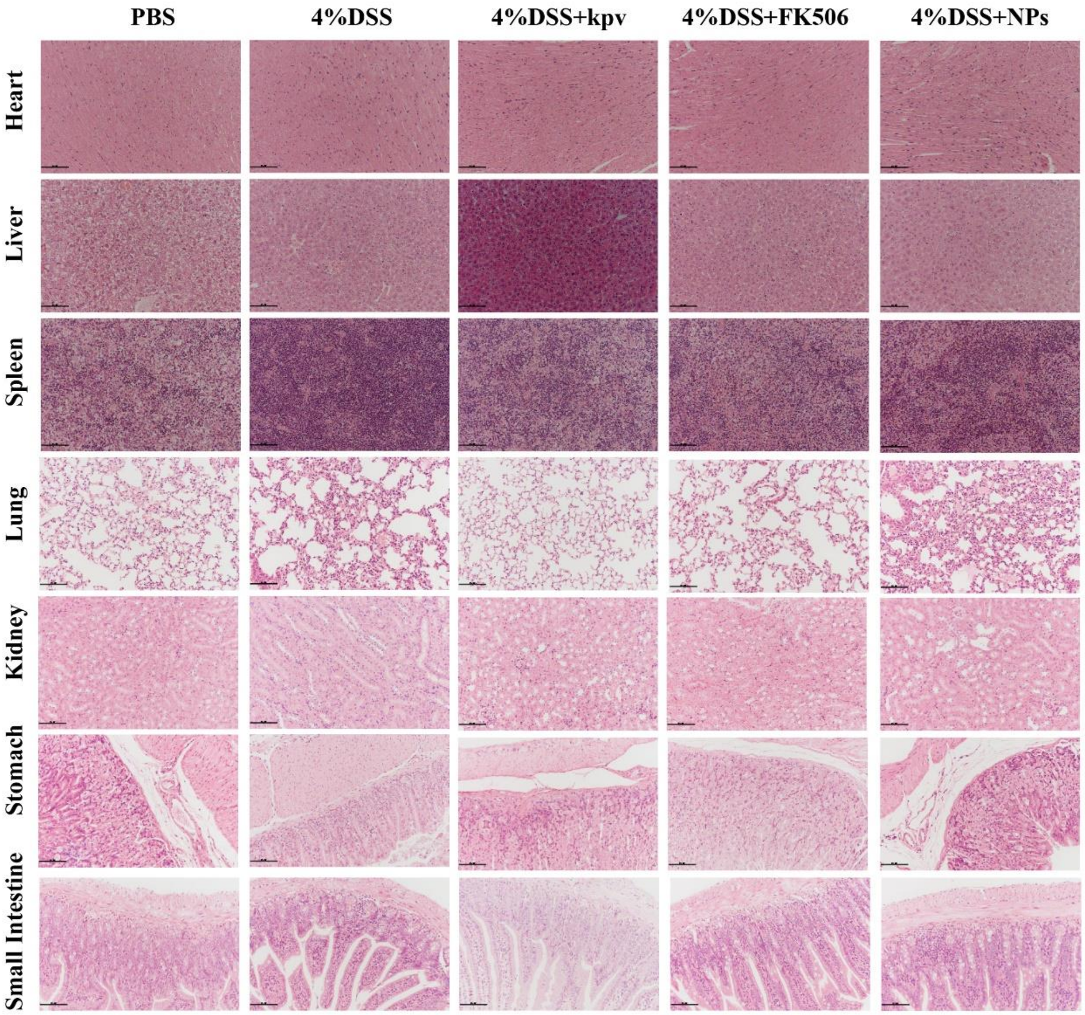

Fig S7. Preliminary biosafety evaluation. The histological examination of the major organs for 4%DSS (scale bar =100 mm). There were no pathological changes. Data are expressed as mean SD.

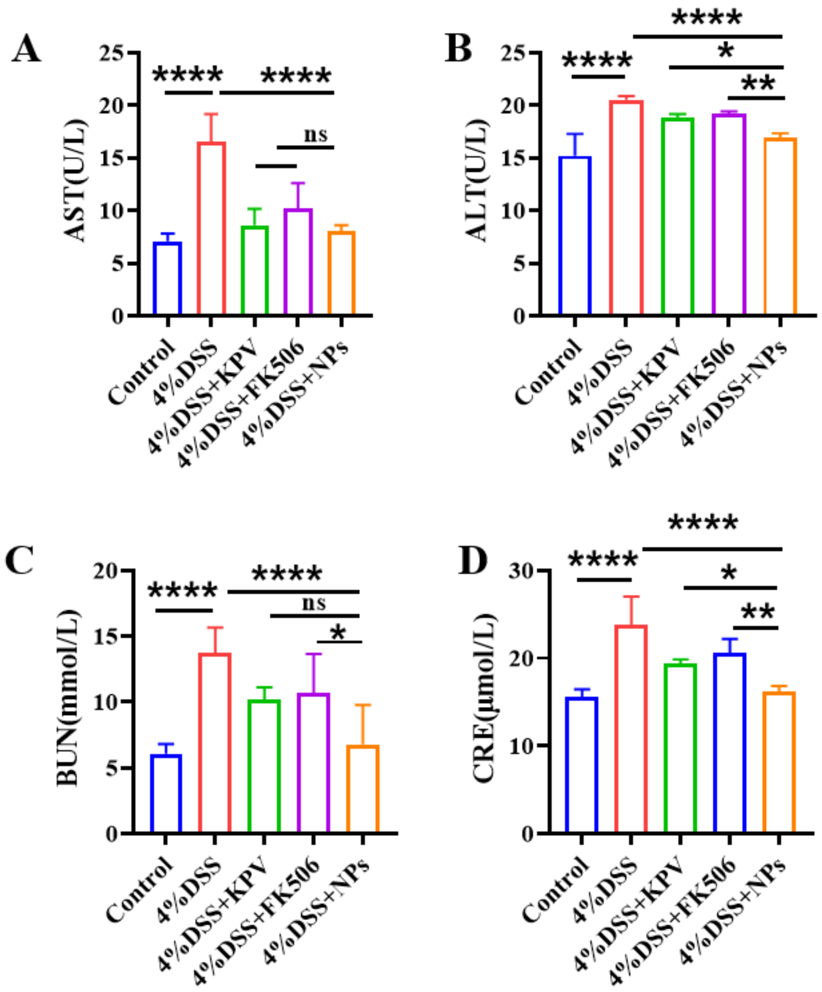

Fig S8. The levels of AST (A), ALT (B), BUN (C) and CRE (D) in serum for 4% DSS. *$P < 0.05$, **$P < 0.01$, ***$P < 0.001$, ****$P < 0.0001$; ns, no significance.

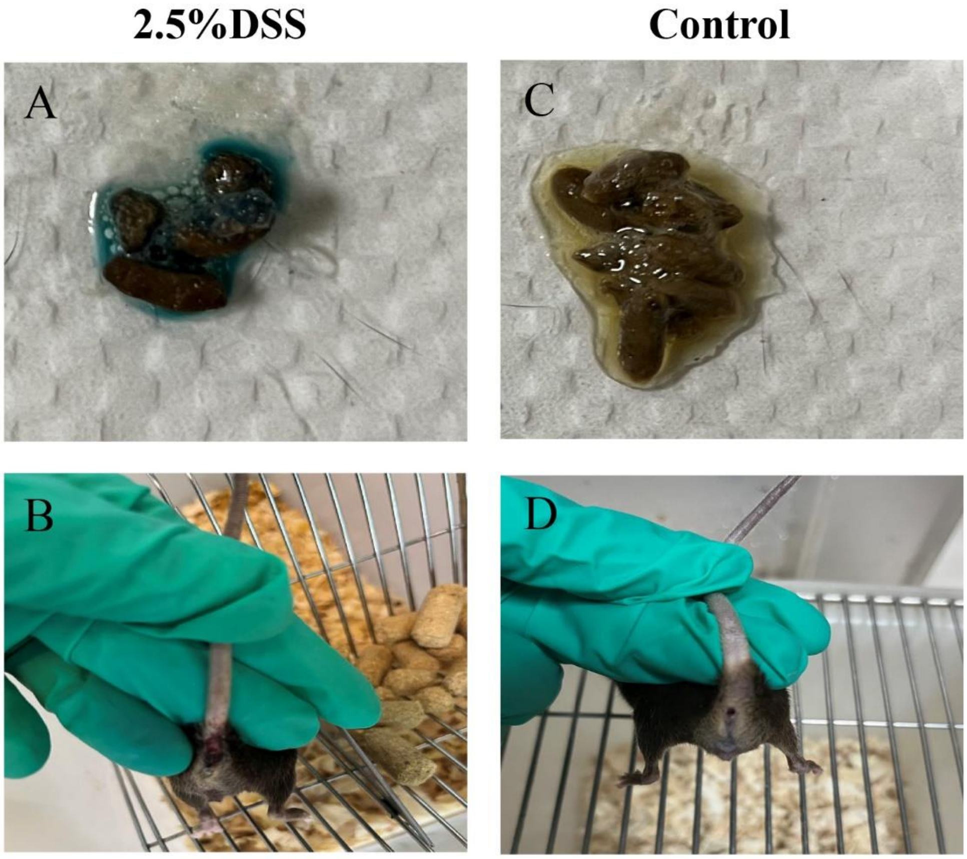

Fig S9. 2.5%DSS experimental group (A), (B) and Occult blood test in control group (C), (D).

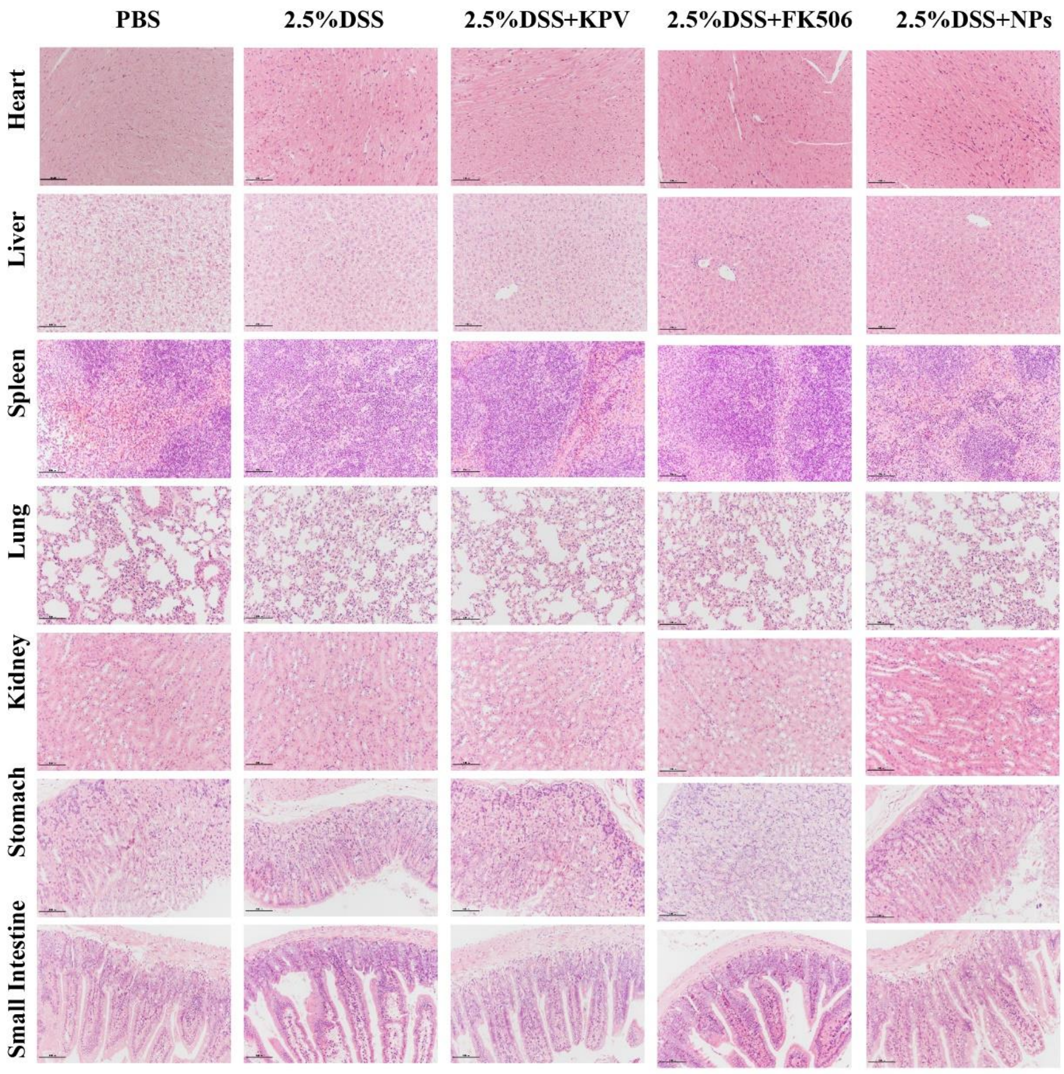

Fig S10. Preliminary biosafety evaluation. The histological examination of the major organs for 2.5%DSS (scale bar =100 mm). There were no pathological changes. Data are expressed as mean SD.

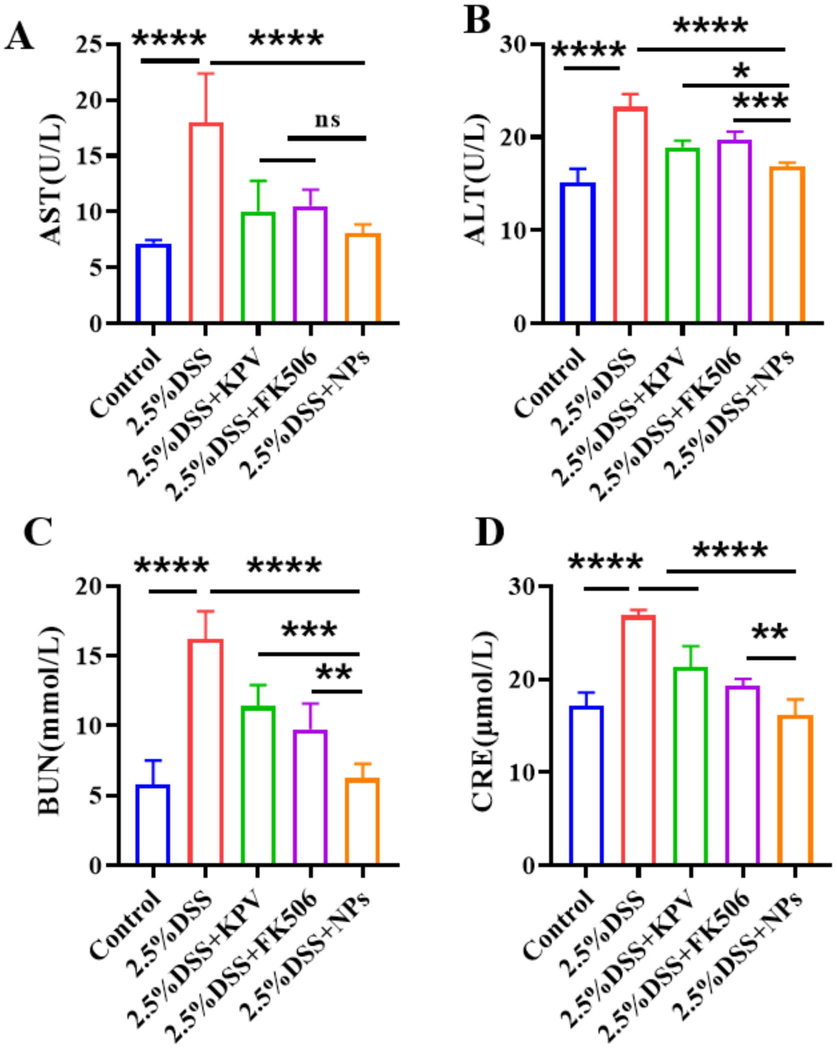

Fig S11. The levels of AST (A), ALT (B), BUN (C) and CRE (D) in serum for 4% DSS. **P* < 0.05, ***P* < 0.01, ****P* < 0.001, *****P* < 0.0001; ns, no significance.

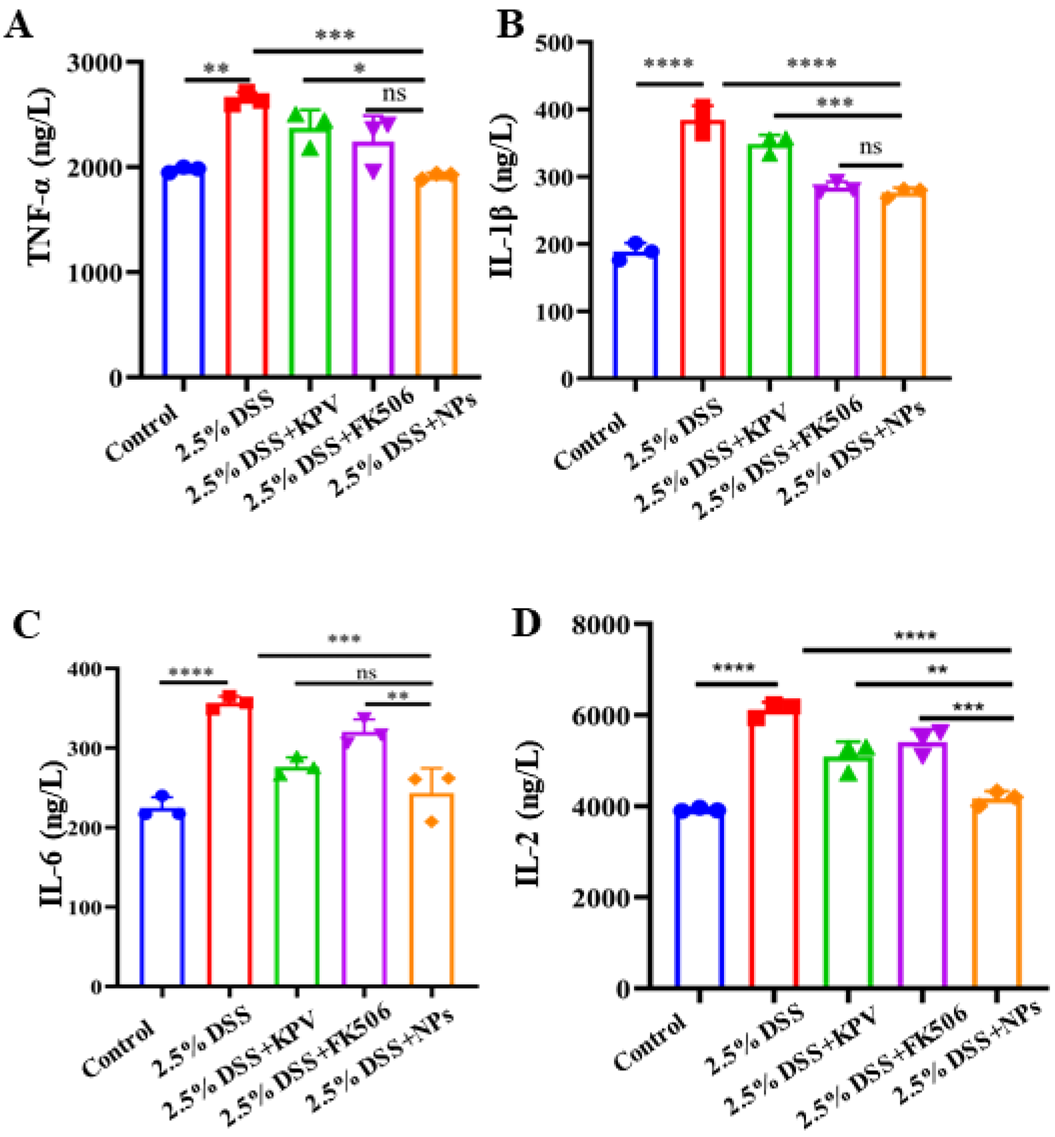

Fig S12. Detection of serum proinflammatory cytokines (A) TNF-α, (B) IL-1β, (C)IL-6, (D) IL-2 in different groups of mice by ELISA. *$*P < 0.05$, $**P < 0.01$, $***P < 0.001$, $****P < 0.0001$*; ns, no significance.

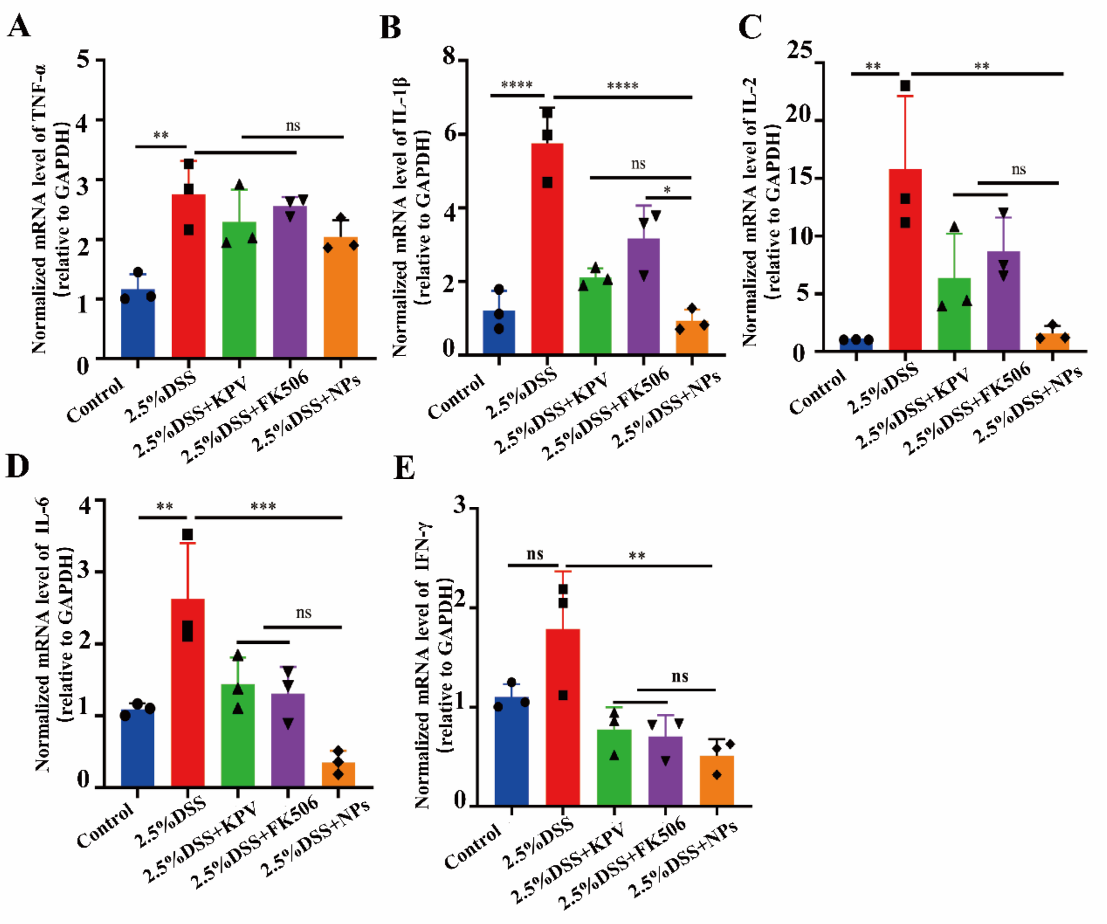

Fig S13. Cytokines (A) TNF-α, (B) IL-1β, (C) IL-2, (D) IL-6 and (E) IFN-γ in the colons detected by qPCR for 2.5% DSS. *$P < 0.05$, **$P < 0.01$, ***$P < 0.001$, ****$P < 0.0001$; ns, no significance.
